# Efficacy and safety of PCSK9 inhibitors, potent statins, and their combinations for reducing low-density lipoprotein cholesterol in hyperlipidemia patients: a systematic network meta-analysis

**DOI:** 10.3389/fcvm.2024.1415668

**Published:** 2025-02-05

**Authors:** Yuhua Jiang, Yingying Wang, Sijia Ma, Linlin Qian, Yeteng Jing, Xi Chen, Jinsheng Yang

**Affiliations:** ^1^ Institute of Basic Theory of Traditional Chinese Medicine, China Academic of Chinese Medical Sciences, Beijing, China; ^2^ Institute of Acupuncture and Moxibustion, China Academy of Chinese Medical Sciences, Beijing, China

**Keywords:** hyperlipidemia, LDL-C, PCSK9 inhibitors, potent statins, network meta-analysis

## Abstract

**Background:**

The objective of this study is to assess the relative efficacy of proprotein convertase subtilisin/kexin type 9 (PCSK9) inhibitors, such as alirocumab, evolocumab, and inclisiran, in conjunction with potent statins like atorvastatin and rosuvastatin, in patients presenting with hyperlipidemia or heightened cardiovascular risk attributable to elevated low-density lipoprotein cholesterol (LDL-C).

**Methods:**

A systematic search was conducted across databases including PubMed, Embase, and the Cochrane Library to explore lipid-lowering therapies in hyperlipidemia from their inception to 7 November 2023. A network meta-analysis (NMA) was conducted via Stata 17 software, with two authors independently conducting the search, screening, and data abstraction.

**Results:**

A total of 68 clinical studies involving 21,288 patients with hyperlipidemia were incorporated into the NMA. PSCK9 inhibitors and potent statins significantly reduced LDL-C levels from baseline vs. placebo regardless of background therapy. Regarding the efficacy of lipid reduction, four principal medications were evaluated: evolocumab and atorvastatin [mean standard deviation (MD) −3.41, 95% CI −4.81 to −2.00] and evolocumab with rosuvastatin (MD −3.44, 95% CI −5.10 to −1.78) vs. placebo; alirocumab combined with rosuvastatin (MD −2.91, 95% CI −3.95 to −1.88) and alirocumab with atorvastatin (MD −2.90, 95% CI −3.97 to −1.84) vs. placebo. Meanwhile, compared with placebo, evolocumab (MD −1.89, 95% CI −2.27 to −1.50), alirocumab (MD −1.83, 95% CI −2.09 to −1.57), rosuvastatin (MD −1.93, 95% CI −2.30 to −1.56), inclisiran (MD −1.68, 95% CI −2.10 to −1.27), and atorvastatin (MD −1.68, 95% CI −2.04 to −1.31) could also play a role in the treatment of LDL-C reduction. Moreover, the incidence of adverse events (AEs) was similar to that observed in the control group, which included both placebo and potent statin groups, with no significant differences identified in our study (*P* > 0.05).

**Conclusions:**

The combination of PCSK9 inhibitors with robust statins like rosuvastatin and atorvastatin markedly decreases LDL-C levels in patients with hyperlipidemia when compared to placebo or monotherapy. Notably, the pairing of evolocumab and atorvastatin exhibited exceptional efficacy in this investigation. In the interim, the combination of PCSK9 inhibitors and potent statins demonstrates a notable safety profile when contrasted with the control group.

## 1 Introduction

Low-density lipoprotein cholesterol (LDL-C) is an important biomarker of the body's glycolipid metabolism and has been linked to an elevated risk of significant cardiovascular events such as atherosclerosis. An elevated LDL cholesterol level has become the third biggest risk factor that leads to cardiovascular disease except for high blood pressure and high-sodium diet (1). The recent 2023 ESC guidelines recommend that the goal of LDL-C should be <55 mg/dl (<1.4 mmol/L) and >50% LDL-C reduction from baseline in patients with diabetes and lower-extremity artery disease (LEAD) at extremely high cardiovascular risk (CV) (2). Hypercholesteremia is a major part of hyperlipidemia including high low-density lipoprotein in atherosclerotic cardiovascular disease (ASCVD) and HeFH, which have an excess risk of cardiovascular mortality and eating disorders (3, 4). Therefore, lowering LDL-C is the primary treatment of hyperlipidemia and cardiovascular disease to prevent other serious complications.

Currently, statins are recognized as the fundamental and efficacious treatment for lowering LDL cholesterol levels in cases of atherosclerotic cardiovascular disease (ASCVD), contributing to a reduction in both mortality and morbidity. This is particularly true for certain potent statins, such as atorvastatin and rosuvastatin (5). However, some patients may not achieve standard LDL-C values through common statin monotherapy and cannot tolerate statins at higher intensities, even some that are intolerant entirely (6, 7). Thus, high-intensity statins alone may not be enough in some patients. The 2022 ACC Expert Consensus suggests that statins can be supplemented by other lipid-lowering agents, such as proprotein convertase subtilisin/kexin type 9 inhibitors (PCSK9i), bempedoic acid, or ezetimibe, for further optimizing LDL-C reduction and managing LDL-related ASCVD risk (8). Meanwhile, the new recommendations in the 2023 ESC guidelines are that PCSK9 inhibitors being advised for patients at extremely high CV risk with persistently elevated LDL-C levels, despite treatment with the highest tolerated statin dose plus ezetimibe, or patients with statin intolerance.

PCSK9 inhibitors are a new class of potent medications that lower cholesterol levels by specifically targeting LDL receptors and enhancing their clearance. These drugs are increasingly being used in patients with high cardiovascular risk and hyperlipidemia (9, 10). For example, certain emerging medications such as alirocumab, evolocumab, and inclisiran are now being administered and researched in clinical trials to lower LDL levels. These medications work by either blocking the binding of PCSK9 to the low-density lipoprotein receptor (LDLR) or inhibiting the production and translation of PCSK9 (11, 12). In addition, most agents have shown significant efficacy in facilitating plaque regression for patients with familial hypercholesterolemia and high cardiovascular risk (13). PCSK9 inhibitors and statins are principal agents for treating coronary atherosclerosis with cholesterol-lowering effects and anti-inflammatory as well as plaque-stabilizing properties (14). Such medications not only lower lipid levels but also protect vascular function, thereby reducing the incidence of cardiovascular events and expanding clinical applications.

Therefore, we conduct a systematic review and network meta-analysis (NMA) to provide a detailed assessment of the efficacy of PCSK9 inhibitor agents such as alirocumab, evolocumab, and inclisiran and powerful statins including atorvastatin and rosuvastatin for reducing LDL-C in patients with hyperlipidemia.

## 2 Methods

### 2.1 Literature search and study selection

To identify all available randomized, controlled trials (RCTs) for evaluating the efficacy of PCSK9 monoclonal antibodies and potent statins in hyperlipidemia, we searched databases including PubMed, Embase, and the Cochrane Library from their inception to 7 November 2023. Meanwhile, the trial registers in ClinicalTrials.gov were also sought as supplements, and the articles published in English were selected. Detailed information regarding the search strategy can be located in Supplementary Table S1.

The following are the inclusion/exclusion criteria for the systematic review according to the PICO principle:

Inclusion criteria:

(1) Population—all patients with hyperlipidemia and high LDL levels, with no age restriction.

(2) Intervention: the treatment group includes alirocumab, evolocumab, inclisiran, atorvastatin, rosuvastatin alone, and their combination. The control group is placebo or statins.

(3) Outcome: LDL-C level reduction, adverse events (AEs).

(4) Study design: a randomized controlled trial at least 4 weeks in duration.

(5) The article was published in English.

Exclusion criteria: The study that was not available, lack of relevant outcome data, animal or cell research, treatment course was less than 4 weeks, and other network meta-analysis were excluded.

Our researchers performed an initial screening by evaluating the titles and abstracts. Additionally, we eliminated replicated and inaccessible research, as well as studies conducted on animals or cells. We also deleted studies that did not meet the criteria of randomized controlled trials. Furthermore, we excluded studies that focused on alternative lipid-lowering treatments such as ezetimibe, bempedoic acid, and other anti-PCSK9 antibodies such as bococizumab, among others. Then, the full-text articles of these studies were assessed again for the second screening. Finally, we would discuss and resolve it if some disagreements occur in the screening process.

### 2.2 Data collection and quality assessment

Two writers autonomously extracted all data, including the research name, year of publication, population, sex, age, treatment duration, and interventions of trials, based on the original publication. Another reviewer conducted a thorough examination of the data to identify any errors. The examination was based on specific criteria established in advance. These criteria were designed to identify randomized controlled trials (RCTs) that compared the effectiveness of alirocumab, evolocumab, and inclisiran, either alone or in combination with atorvastatin or rosuvastatin, against a placebo or another drug. The trials focused on patients with hyperlipidemia, including those with hypercholesterolemia, heterozygous familial hypercholesterolemia (HeFH), homozygous familial hypercholesterolemia (HoFH), ASCVD, and dyslipidemia, who were at risk of cardiovascular problems due to inadequately controlled LDL-C levels.

The type of study included populations was not restricted, and the LDL levels were significantly increased at baseline. Typically, we employed GetData software to extract LDL-C data from diagrams and graphs in the absence of percentage changes via text or the Cochrane assessment tool. Meanwhile, the RevMan 5.3 software was also used to conduct risk of bias in all trials including random sequence generation, random concealment, blinding of participants and personnel, blinding of outcome assessment, incomplete outcome data, selective reporting, and other biases.

### 2.3 Data analysis and synthesis

The network meta-analysis (NMA) represents a sophisticated statistical methodology designed to elucidate variations in treatment effects by integrating both direct comparisons and numeric absolute values within the article. Meanwhile, the risk of bias was apparent in indirect evidence (15). In this essay, we employed NMA to synthesize and analyze the results of the included clinical trials and estimate the efficacy of PCSK9 inhibitors and potent statins vs. placebo. The Stata 17.0 (Stata corporation, USA) containing mvmeta and network that were used to draw the trial network plots and assess for publication bias. The mean standard deviation (MD) was employed as a conventional metric for continuous outcomes, while the odds ratio (OR) was utilized for dichotomous variables. We establish a confidence interval of 95% to evaluate outcome measures and determine a threshold for statistical significance at *p* < 0.05 for testing purposes. In addition, a representative funnel plot was used to test potential publication bias. To evaluate the heterogeneity and inconsistency among these studies, we conducted the meta-analysis in Stata 17.0 software incorporating the *I*^2^ value. The value of *I*^2^ which is >50% indicated significant heterogeneity between studies, which required the application of a random-effects model. In contrast, if the *I*^2^ value was 50% or less, minimal heterogeneity was present, making the fixed-effects model appropriate. The sensitivity and specific heterogeneity analyses was considered in the direct comparisons, and the corresponding results were detailed in the Supplementary material. Meanwhile, the consistency of the study was confirmed when *p* > 0.05, and the test of inconsistency between direct and indirect comparisons was assessed through the node-splitting method.

A frequency framework was employed, and a random-effects model was implemented in the network meta-analysis. Outcome measures such as LDL-C percent change or absolute values at 4–144 weeks were used as inputs to analyze, and the 12-week or 24-week data were most common in this analysis. Furthermore, data regarding adverse events (AEs) were meticulously extracted and analyzed to perform a comprehensive safety assessment. Due to the number of trials treatment and heterogeneity in the research, *I*^2^ > 50%, RE models were most appropriate in the LDL-C study. In the process of NMA, the dose of drugs was not restricted grimly. For instance, alirocumab was usually administered at 75 mg every 2 weeks (Q2W) or 150 mg Q2W, evolocumab at 140 mg Q2W or 420 mg monthly (QM), and inclisiran sodium at 300 mg (equivalent to 284 mg inclisiran) as a single-use subcutaneous injection. Meanwhile, atorvastatin was given at ∼10–80 mg every day and rosuvastatin at 10–40 mg a day in these trials. In this investigation, we select the routine standard dosage for our analysis when multiple dosages are available.

## 3 Results

### 3.1 Results of the search

**Figure 1:**
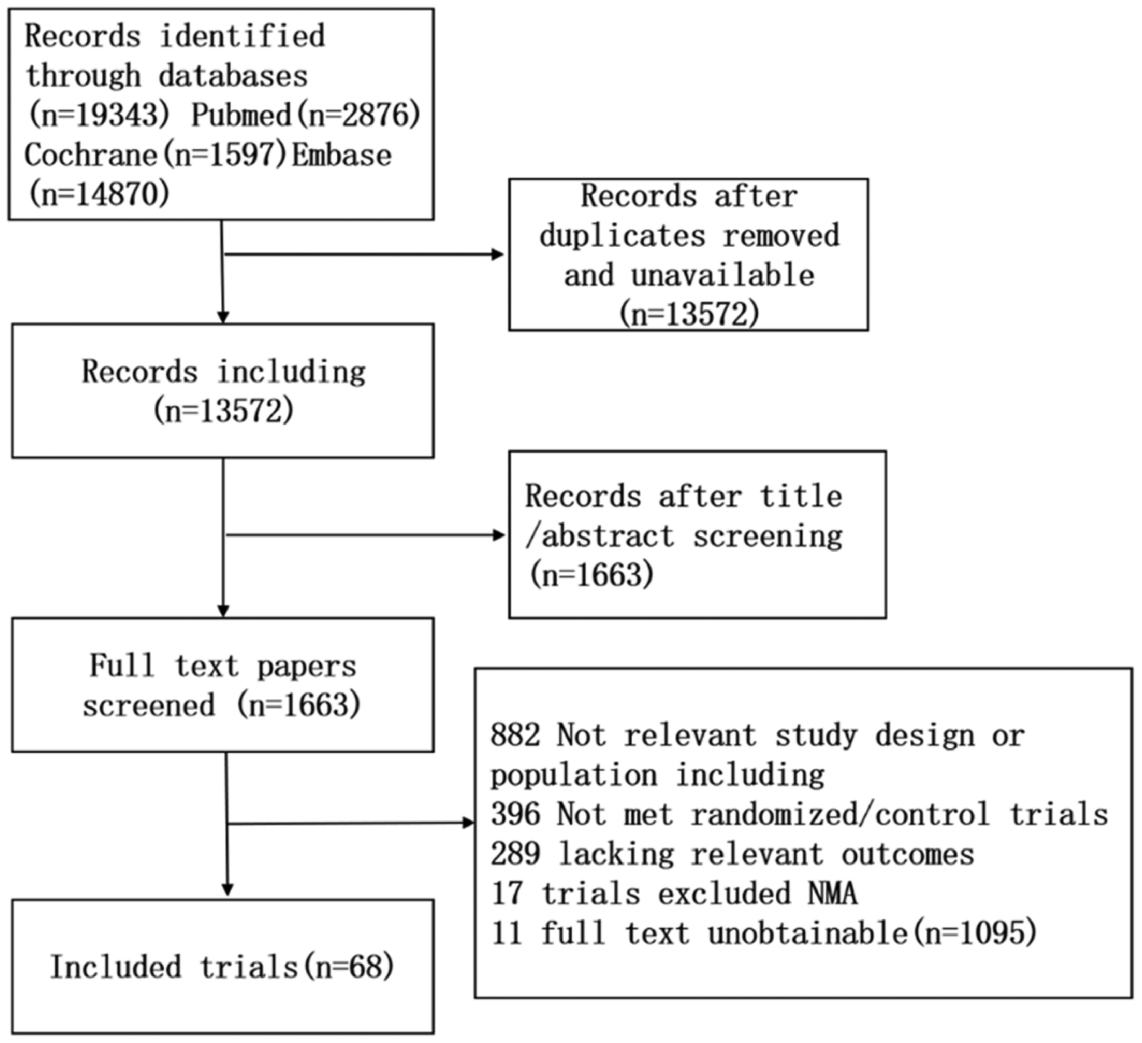
Study flow diagram of the systematic review.

A comprehensive systematic review yielded 19,343 relevant trials based on the established study search strategy. Following the elimination of duplicate records and the meticulous screening of titles and abstracts, 1,163 studies remained for further consideration. Then, 1,163 studies were included through screening full texts, and 68 studies were included in this NMA. The search flow diagram is shown in Figure 1.

### 3.2 Characteristics of the included studies

Supplementary Table S2 delineated the characteristics of the trials incorporated. The published papers examined in this analysis spanned from 1995 to 2023, and the control group consisted of either placebo or statins. Twelve interventions were applied in the 68 randomized clinical trials, including PCSK9 inhibitors, potent statins, and placebo. The duration ranged between 4 and 72 weeks.

### 3.3 Risk of bias assessment

**Figure 2:**
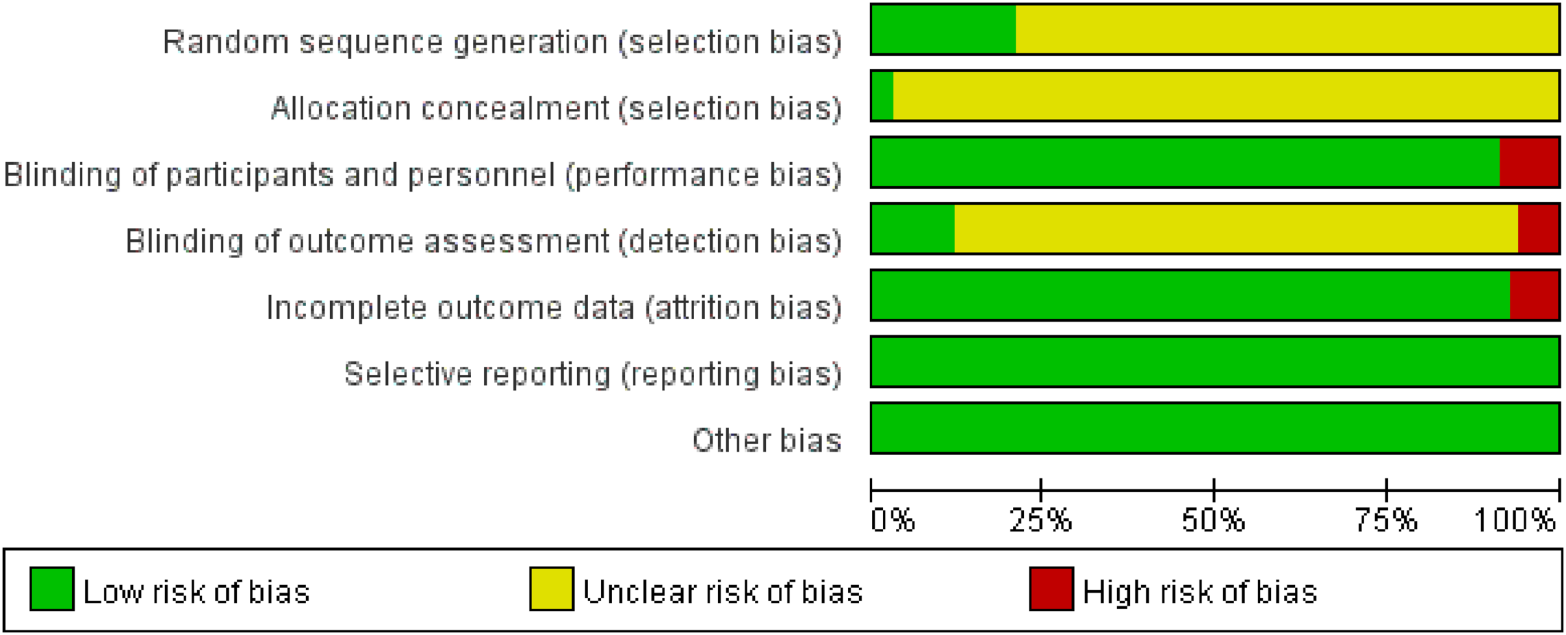
Risk of bias assessment in all included trials.

Of the 68 studies analyzed, 14 trials demonstrated a negligible risk of bias, while 54 presented an ambiguous risk concerning the methods of sequence generation. Two trials exhibited a minimal risk, while others presented an ambiguous risk concerning allocation concealment. In terms of blind methods, all trials had double-blinding of participants and personnel except six trials that were single-blinding or open-label. There were five incomplete outcome data in elevated risk in all included trials, and all randomized clinical trials had a minimal risk of selective reporting bias and other biases. The complete risk assessment is presented in Figure 2.

### 3.4 Presentation of network structure

**Figure 3:**
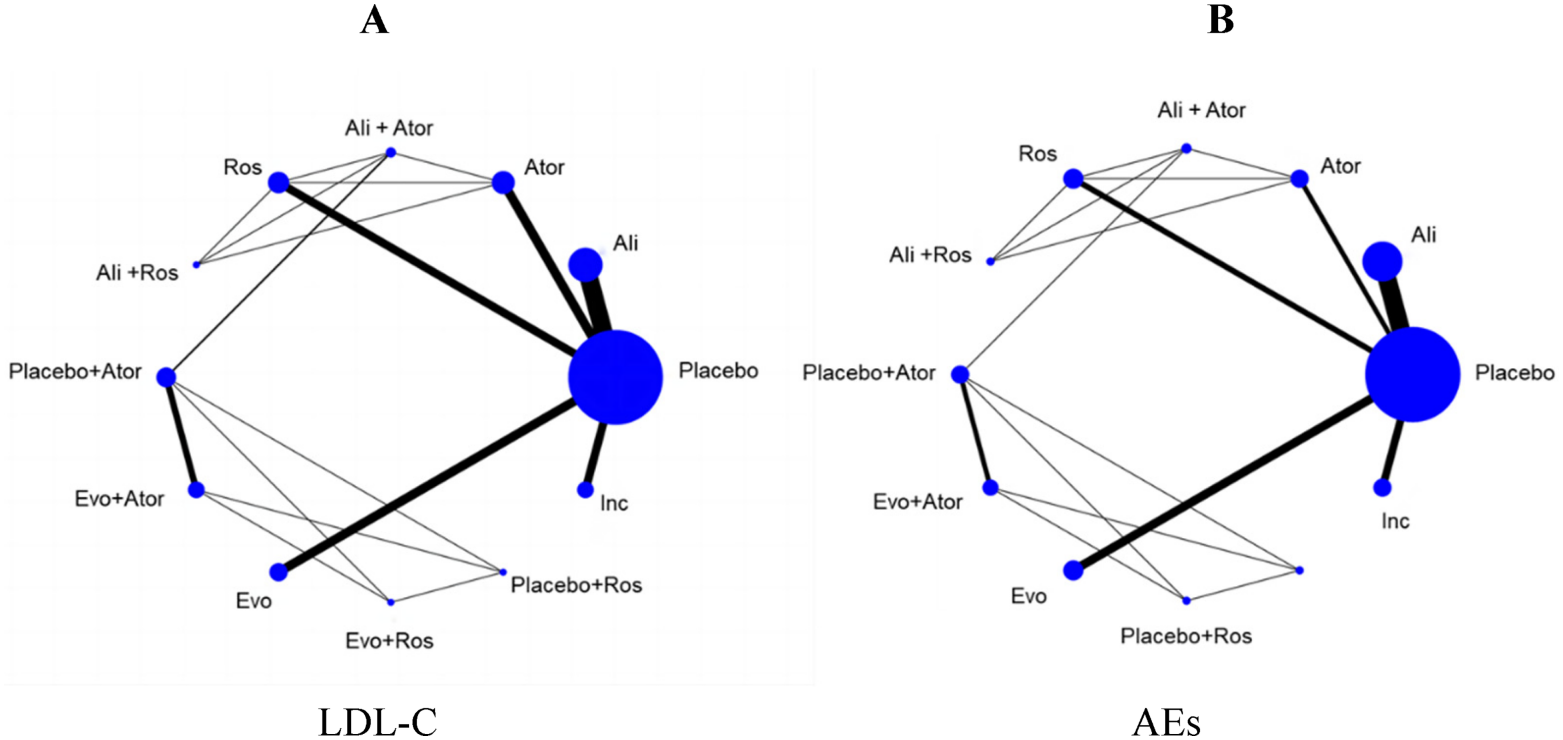
The evidence network diagram of different interventions for hyperlipidemia patients **(A)** LDL-C levels. **(B)** Adverse events. Ali, alirocumab; Ator, atorvastatin; Ros, rosuvastatin; Evo, evolocumab; Ins, inclisiran. Ali+Ator, alirocumab plus atorvastatin; Ali+Ros, alirocumab plus rosuvastatin; Placebo+Ator, placebo plus atorvastatin; Placebo+Ros, placebo plus rosuvastatin; Evo+Ator, evolocumab plus atorvastatin; Evo+Ros, evolocumab plus rosuvastatin.

The network structure for this meta-analysis for LDL-C reduction is shown in Figure 3A. The size of the treatment nodes corresponded to the number of hyperlipidemia patients, and the thickness of the line indicated the number of trials comparing several kinds of agents in the treatment group. The highest samples in this NMA were placebo, including 7,427 patients, and the closest link was alirocumab and placebo, with 20 studies providing data for its comparison. Specifically, there were 23 studies of alirocumab (16–38) and 17 studies of evolocumab (39–55) in the entire network, which included alirocumab or evolocumab combined with atorvastatin and rosuvastatin (20–22, 39–42, 45, 46, 48, 51, 55). Meanwhile, inclisiran (56–64), atorvastatin (65–74), and rosuvastatin (75–83) compared with placebo were also analyzed in the network. Furthermore, the network architecture for AEs was illustrated in Figure 3B, encompassing 56 studies and 12 interventions within the research framework.

### 3.5 Inconsistency and heterogeneity assessment

The inconsistency test between direct and indirect evidence was conducted by using the node-splitting analysis, and the results showed there was no statistical difference (p > 0.05) between whole interventions inconsistency evidence (Supplementary Table S3, Figure S1). The test of heterogeneity among the multiple interventions was presented in Supplementary Table S4. Moreover, we conducted heterogeneity and sensitivity analyses to ascertain the sources of variability in the direct comparison measures through the utilization of forest plots. The findings indicated that the primary outcome, LDL-C levels, exhibited minimal variation in the sensitivity analysis (Supplementary Figures S2, S3).

### 3.6 Publication bias assessment

**Figure 4:**
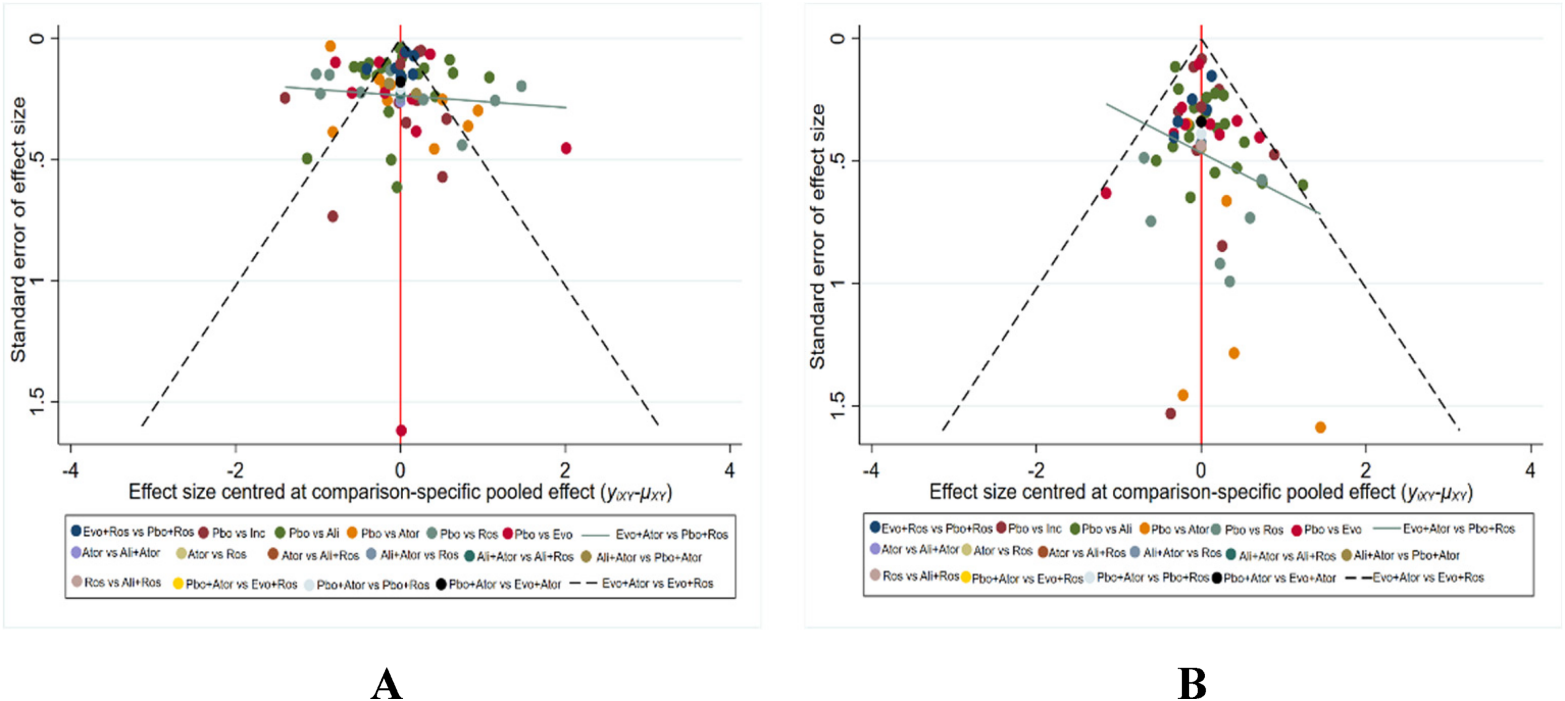
Forest plot for results of outcome indicators LDL **(A)** and adverse drug events **(B)**.

The primary outcome indicator was LDL-C in all treatment trials, and any adverse events (AEs) in treatment were secondary. The bias assessment of different interventions was conducted in terms of LDL level decreasing and the incidence of adverse events. Various agents were represented by distinct colors, with each dot indicating the included trials in Figures 4A,B. This depiction revealed a balanced symmetry in the comparison-specific funnel chart, illustrating a reduced presence of publication bias.

### 3.7 Synthesis of results

#### 3.7.1 All measures in LDL-C reduction

**Figure 5:**
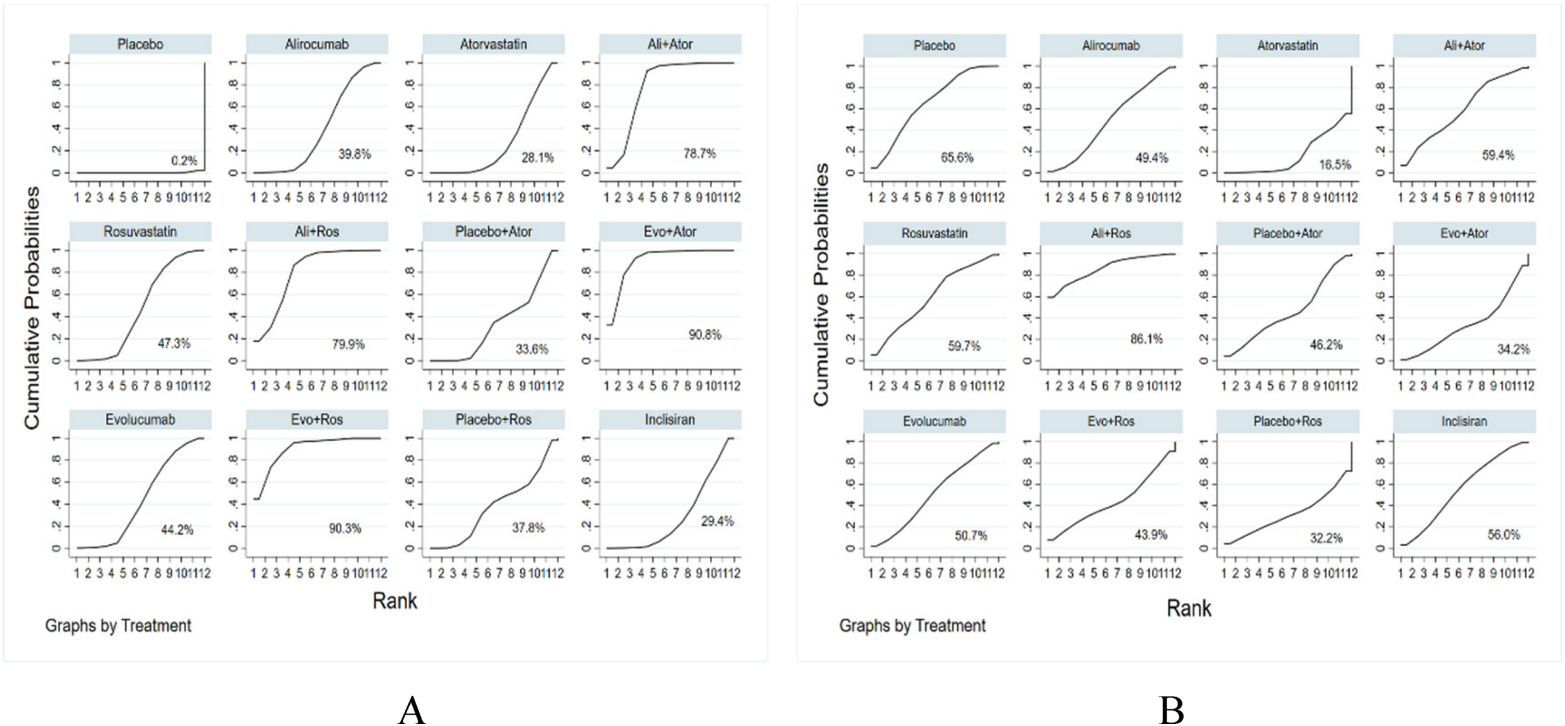
Ranking of treatment strategies based on the probability of their LDL-C reducing **(A)** and adverse drug events reducing **(B)**.

All 68 included studies reported reductions of LDL-C levels as the primary efficacy outcome. The effect of all treatments was ranked with SUCRA probabilities (Figure 5). According to the data analysis, evolocumab together with atorvastatin has the greatest probabilities (SUCRA 90.8%) for the best treatment on reducing LDL levels, closely followed by evolocumab combined with rosuvastatin (SUCRA 90.3%), and next with alirocumab plus rosuvastatin (SUCRA 79.9%), alirocumab with atorvastatin (SUCRA 78.7%), rosuvastatin (SUCRA 47.3%), evolocumab (SUCRA 44.2%), alirocumab (SUCRA 39.8%), placebo with rosuvastatin (SUCRA 37.8%), placebo with atorvastatin (SUCRA 33.6%), inclisiran (SUCRA 29.4%), atorvastatin (SUCRA 28.1%), and placebo (SUCRA 0.2%).

#### 3.7.2 The NMA comparisons for primary efficacy

**Figure 6:**
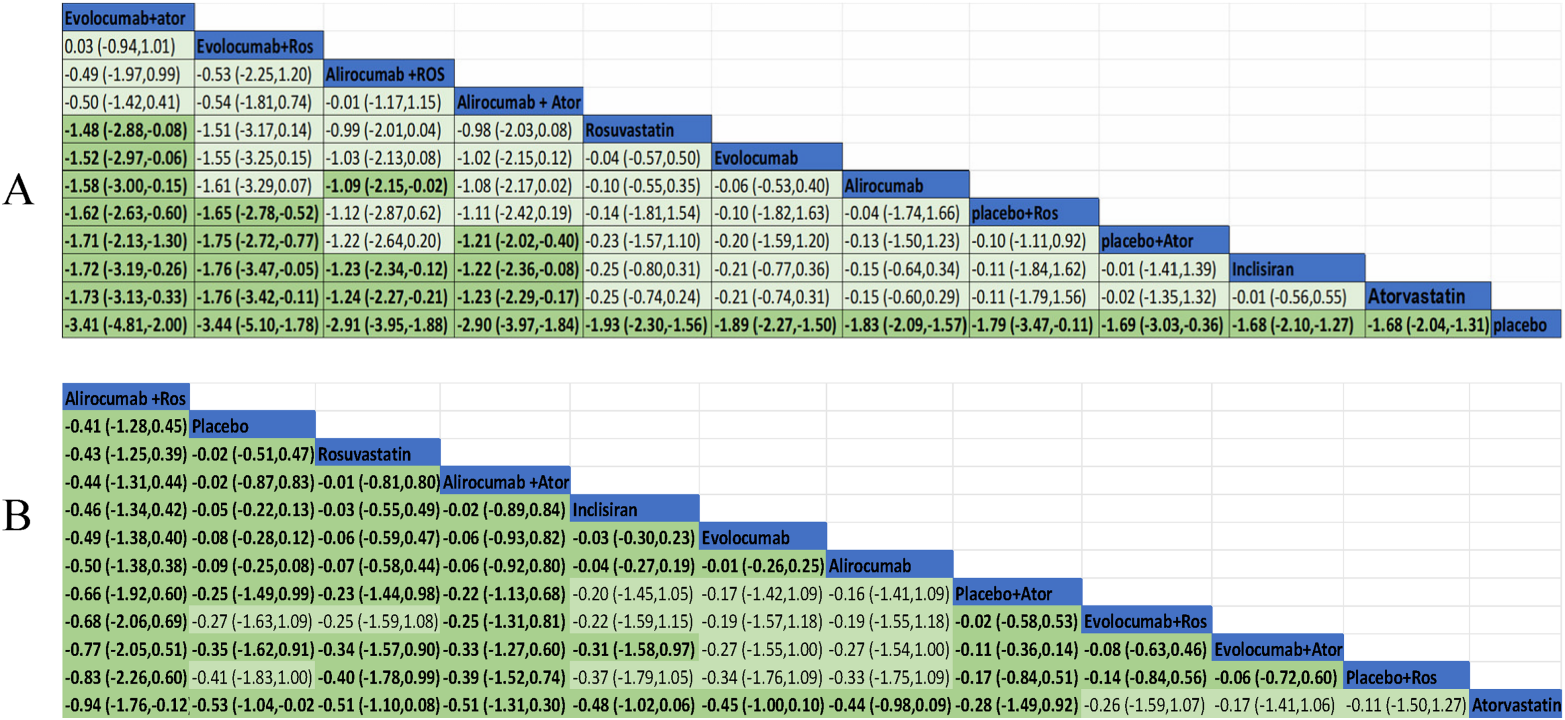
Treatment efficiency comparisons for LDL-C lowering **(A)** and adverse events reactions **(B)**.

We performed a pairwise comparison among several medications in the network to assess their effectiveness in decreasing LDL-C levels. A significant divergence was observed among the pharmaceuticals highlighted in the chart (Figure 6A). The results of the analysis had shown also that LDL-C reduction was more significant in evolocumab combined with atorvastatin compared with evolocumab, rosuvastatin, alirocumab, and inclisiran. Meanwhile, it was greater for evolocumab with rosuvastatin compared with atorvastatin, inclisiran, and placebo plus atorvastatin or rosuvastatin in the reduction of LDL level. Alirocumab with rosuvastatin was more efficient than alirocumab alone and inclisiran as well as atorvastatin. Alirocumab combined with atorvastatin was superior to atorvastatin, inclisiran, and placebo with atorvastatin. All medications demonstrated a markedly greater efficacy in comparison to the placebo. Nevertheless, the other treatment comparisons did not demonstrate significant reductions in LDL-C levels.

#### 3.7.3 Effect of PCSK-9 inhibitors and combination therapy on LDL-C reducing

Figure 6A exhibited the MD and 95% confidence interval (CI) of three lipid-lowing medicines compared with placebo or statin. As shown in the data analysis, evolocumab plays a prominent role in efficiency (MD −1.89, 95% CI −2.27 to −1.50) compared with placebo. Meanwhile, LDL-C was also markedly reduced in treatment with alirocumab (MD −1.83, 95% CI −2.09 to −1.57) and inclisiran (MD −1.68, 95% CI −2.10 to −1.27).

Ten trials were conducted to assess the efficacy of combining PCSK-9 inhibitors with strong statins, specifically alirocumab and evolocumab with atorvastatin or rosuvastatin. In this study, it was found that the combination of evolocumab and atorvastatin is more effective in reducing LDL-C levels compared to atorvastatin alone (mean difference −1.73, 95% CI −3.13 to −0.33). Similarly, the combination of evolocumab and rosuvastatin was more effective in reducing LDL-C levels compared to rosuvastatin alone (mean difference −1.48, 95% CI −2.88 to −0.88). The combination of alirocumab and rosuvastatin was also slightly effective but no statistically significant difference in reducing LDL-C levels compared to rosuvastatin alone (mean difference −0.99, 95% CI −2.01 to 0.04). The synergistic effect of alirocumab in conjunction with atorvastatin demonstrated superior efficacy in lowering LDL-C levels when contrasted with atorvastatin administered in isolation (mean difference −1.23, 95% CI −2.29 to −0.17).

#### 3.7.4 Safety evaluation

The evidence network is illustrated in Figure 3B, encompassing 12 interventions and 18,482 patients, along with 10 direct comparisons within the study. Moreover, we conducted a traditional pairwise meta-analysis regarding the incidence of total adverse events [except for three studies (20, 22, 51) that included only one reference]. The forest plot of direct comparisons illustrated that there were no statistically significant differences in the risk of any AEs between the treatment group and the control group (Supplementary Figure S4). Simultaneously, a network meta-analysis on the incidence of AEs was performed, containing 66 pairwise comparisons. The SUCRA ranking results showed that alirocumab combined with rosuvastatin was the best intervention in terms of reducing the incidence of AEs (Figure 5B). The rank of probability is Ali + Ros > Placebo > Rosuvastatin > Ali + Ator > Inclisiran > Evolocumab > Alirocumab > Placebo + Ator > Evo + Ros > Evo + Ator > Placebo + Ros > Atorvastatin, and the results revealed that alirocumab plus rosuvastatin was superior to rosuvastatin (OR −4.3, 95% CI −1.25 to 0.39) and alirocumab (OR −0.50, 95% CI −1.38 to 0.38). Alirocumab with atorvastatin was more effective than alirocumab (OR −0.06, 95% CI −0.92 to 0.80) and atorvastatin (OR −0.51, 95% CI −1.31 to 0.30). Evolocumab combined with atorvastatin was better than atorvastatin (OR −0.17, 95% CI −1.41 to 1.06), but there was no statistical difference (Figure 6B).

## 4 Discussion

LDL-C is a vital factor in individuals with hyperlipidemia and serves as a significant risk indicator for managing lipids in patients with cardiovascular disease. A reduction of 1 mmol/L in LDL-C can lead to a 21% decrease in the risk of major vascular events according to research (3, 84). Statin drugs are commonly used in clinical practice for lipid-lowering, with a high utilization rate, proven efficacy, and good safety profile. The mechanisms of statin agents exhibit considerable diversity. The synthesis of cholesterol in the liver can be diminished by inhibiting the rate-limiting enzyme involved in its production (HMG-CoA reductase). This action results in an increased presence of low-density lipoprotein receptors on the liver surface, thereby regulating the levels of LDL-C (85). Currently, statin therapy remains the cornerstone of lipid-lowering treatments to prevent ASCVD, which is also essential to maintain continuous therapy even though there is partial statin intolerance clinically. The guidance from the United States suggests that adjusting the statin dosage to enhance tolerability or incorporating non-statin medications may constitute an effective therapeutic approach (86). Conversely, a considerable number of patients continue to attain therapeutic efficacy with a specific dosage of statin, owing to the notable variability in statin intolerance observed currently, with instances of complete intolerance being exceedingly rare, occurring in fewer than 5% of cases (87). Research in clinical settings is persistently advancing, contributing to a more profound comprehension of these subjects. Among statins, atorvastatin and rosuvastatin belong to the same type of drugs, and their application rates are steadily increasing among various drugs. They have exhibited similar effectiveness regarding the combined outcome of all-cause death, myocardial infarction, or coronary revascularization during a 3-year period in coronary artery disease (88).

Nevertheless, in specific circumstances, there were instances of adverse reactions that could be linked to the consumption of high doses of statin medications, surpassing the patient's tolerance, including hepatotoxicity and rhabdomyolysis, which exhibit a dose-dependent relationship (89, 90). The occurrence of new-onset diabetes mellitus or glucose intolerance has been rising as a side effect of statin therapy through aggravating insulin resistance (91). At the same time, the included literature data showed that statin-related adverse events primarily consisted of headaches, insomnia, gastrointestinal issues, etc. in our research. In recent years, several non-statin lipid-lowering therapies such as PCSK9 inhibitors, ezetimibe, and bempedoic acid could be effective in considerable-risk CV patients who are unable to tolerate maximal statin. Numerous combinations have been demonstrated to optimize LDL-C levels and reduce the risk of ASCVD, supported by evidence from clinical trials (92). In pertinent meta-analyses, statins demonstrated a reduction in LDL-C by an average of 39% and significantly decreased levels of apolipoprotein B (apoB) and triglycerides (93). Meanwhile, the 2022 ACC Consensus indicated that the combination of ezetimibe with statins could yield an additional average reduction in LDL-C levels of 20%–25%; conversely, PCSK9 inhibitors can decrease LDL-C levels by an average of 60%. Thus, PCSK9 inhibitors can decrease LDL-C levels and mitigate ASCVD risk when used with statin therapy, and they are being explored as innovative agents for lipid reduction.

PCSK-9 inhibitors, a newer expensive drug in clinical trials, are one of the effective pathways for lipid-lowering. PCSK9 is a serine protease primarily expressed in the liver. The low-density lipoprotein receptor (LDLR) is the primary receptor responsible for the uptake of cholesterol by peripheral cells. Approximately 75% of cholesterol in circulation is taken up and broken down by the process of LDLR endocytosis. PCSK9 inhibitors facilitate a decrease in LDL levels by obstructing the degradation of LDLR mediated by the PCSK9 protein, thereby enhancing the expression of LDLR on cellular surfaces (94, 95). Meanwhile, PCSK9 levels in plasma are linked with atherosclerosis development by lipid pathways as a promising biomarker in atherosclerosis. Previous research indicates that PCSK9 inhibitors may reestablish the equilibrium among plasma PCSK9, LDL-C, and LDLR, thereby safeguarding vascular function in response to elevated PCSK9 levels in the plasma of individuals with HeFH following treatment with high-efficacy statins and ezetimibe (96). PCSK9 inhibitors have the potential to decrease low-density lipoprotein and cholesterol levels while also safeguarding vascular function, which may contribute to a reduction in the incidence and mortality associated with cardiovascular events to some extent. Currently, three PCSK-9 inhibitors in this research were administered via subcutaneous injection every 2 weeks or once a month, which greatly facilitated the treatment for patients. PCSK9 inhibitors may be a better choice for high-risk cardiovascular patients who still cannot achieve target LDL levels after high-dose potent statin therapy or who cannot tolerate statin drugs in clinical.

We conducted a network meta-analysis of the efficiency of 12 interventions for hyperlipidemia treatment. There were direct and indirect comparisons in the network displayed. PCSK9 inhibitors were proven highly effective for hypercholesterolemia and atherosclerotic cardiovascular disease in a previous meta-analysis (97). Atorvastatin and rosuvastatin were two conventional and regular medications with extensively documented hypolipidemic effects. A recent NMA has shown that combination therapy represents a consistently effective approach to reducing LDL-C levels, particularly through the integration of evolocumab and alirocumab with maximally tolerated statins (98). Meanwhile, a systematic review regarding familial hypercholesterolemia manifested that there was high-quality evidence indicating that alirocumab and evolocumab were effective and safe in LDL-C lowering and almost unchanged neuronal events vs. the placebo. They also significantly lowered LDL levels and reduced coronary allograft vasculopathy after heart transplantation, as well as demonstrated the long-term safety of PCSK9 inhibitors in another meta-analysis (99, 100). In recent systematic reviews and meta-analyses, it was evident that comparisons of PCSK9i have been made with placebo or statin plus ezetimibe, rather than between the three specific inhibitors combined with common statins and single PCSK9i or statins. Also, most NMA studies focused on a single type of dyslipidemia, which partially limited the research range. Correspondingly, our network was built including direct and indirect comparisons to conduct the meta-analysis, which had smaller publication bias with systematic search methods and selection criteria. The results of the sensitivity analysis revealed that there was no significant change in the ranking of PCSK9 inhibitors and statin intervention after sensitivity analyses, which confirmed the primary findings and enhanced the credibility of the NMA results. Another characteristic was that the scope of our research encompassed three significant PCSK9 inhibitors and two widely recognized potent statins, which were representative and had not been the focus of published meta-analyses, thereby excluding other therapies such as ezetimibe, bempedoic acid, and the remaining infrequently utilized statins. At the same time, our research targeted multiple dyslipidemia diseases with elevated LDL-C levels, and treatment focused on several lipid-lowering drugs combination or monotherapy, providing some potential clinical guidance. Nonetheless, the instances of combination therapy were limited due to the scarcity of included studies, resulting in a deficiency of substantial clinical data to underpin our research.

In the present study, the network meta-analysis demonstrated the beneficial effects of alirocumab, evolocumab, inclisiran, rosuvastatin, and atorvastatin and their integration compared with placebo or lipid-lowering medication alone in LDL-C reduction. The data analysis indicated that drug combinations demonstrated greater efficacy in reducing lipid levels compared to individual agents. However, certain comparisons, such as alirocumab combined with rosuvastatin vs. rosuvastatin alone, evolocumab in relation to alirocumab, and inclisiran alongside statins, did not achieve statistical significance. The SUCRA value was the highest in all results on evolocumab plus atorvastatin, followed by evolocumab with rosuvastatin and alirocumab with rosuvastatin. Specifically, evolocumab plus atorvastatin or rosuvastatin was more effective than evolocumab, atorvastatin, and rosuvastatin; alirocumab with atorvastatin or rosuvastatin was also greater than alirocumab or atorvastatin. With respect to drug risk and adverse reactions, there were no significant differences among the various interventions including PCSK9 inhibitors and potent statins in the NMA, and clinical treatment demonstrated greater safety. Nonetheless, the European pharmacovigilance database reported that alirocumab or evolocumab were associated with neurological events and cognitive adverse reactions including headache, insomnia, and depression as the suspected drug (101). Therefore, we specifically conducted a statistical analysis of neurological adverse events, and the results showed that the adverse reaction occurred in the alirocumab and evolocumab treatment group, but statistical analysis showed no significant difference compared to the placebo control. Additionally, neurocognitive events were mentioned in only a few studies, primarily those involving alirocumab treatment in our literature data, but with no significant difference either (Supplementary Figures S4, S5). Furthermore, the latest systematic review of the inclisiran had shown excellent efficacy in managing dyslipidemia that linked to a significant LDL-C decline even if an increased risk of injection site reactions was observed while most of them appeared to be mild and tolerable (102). Hence, PCSK9i had good safety and was suitable in clinical, but this could also be attributed to the limited number of studies included.

There are also some limitations to this review. In our investigation, the NMA was unable to obtain adequate results due to the intricate interplay of various influencing factors. We only evaluated the effects of the primary drug categories in hyperlipidemia treatment instead of conducting some specific analysis based on the drug dose effect, patient populations, intervention duration, etc. For example, it is not possible to provide clear recommendations for the best age and most effective treatment duration for using PCSK9 inhibitors with powerful statins to treat hyperlipidemia owing to the wide age ranges and lengthy treatment durations in these studies. Meanwhile, the clinical classification of hyperlipidemia varied in this research, which contained hypercholesterolemia, HeFH, HoFH, dyslipidemia, and so on, leading to an inconsistent baseline and efficiency in the study. The subgroup analyses involving drug dosages, patient population, and intervention duration failed to clarify the efficacy of various treatments in hyperlipidemia. The presence of heterogeneity is an unavoidable consequence of the intrinsic limitations associated with network meta-analysis methodology, as well as the baseline disparities observed in the original studies, even though we conducted subgroup and sensitivity analyses. Moreover, there has been a notable scarcity of clinical research focusing solely on the use of statins in recent years, coupled with an insufficient body of literature regarding the combination therapy of PCSK9 inhibitors and statins, which may indicate a shortcoming in the current study. Thus, a larger-scale combination of therapy and novel research is needed in this study of lipid-lowering drugs. Furthermore, in this NMA, we concluded that combination therapy (e.g., evolocumab with atorvastatin) was superior in reducing LDL-C but neglected the clinical limitations caused by the cost-effectiveness and accessibility barriers of the drug. For instance, PCSK9 inhibitors plus statin had a higher cost compared to high-dose statin and ezetimibe plus statin in a recent model cost study (103). Moreover, most PCSK9i medicine was provided via subcutaneous injections which could be inconvenient in the daily treatment of patients. There are no major advantages of injectable PCSK9i drugs in terms of ease of administration, cost, or patient preference. However, the Chinese healthcare system is making all efforts to reduce the annual cost of PCSK9 inhibitors, and the price of alirocumab and evolocumab has been reduced by approximately 70% (104). Consequently, the combination of PCSK9 inhibitors and statins demonstrates advantageous therapeutic outcomes in clinical practice. Despite encountering certain transient challenges related to cost efficiency and patient adherence, this approach continues to offer considerable promise and importance for sustained treatment.

## 5 Conclusion

In summary, the network meta-analysis showed that alirocumab, evolocumab, inclisiran, atorvastatin, rosuvastatin, and their combination could significantly reduce LDL-C levels in hyperlipidemia patients. Our research indicated that the combination of PCSK9 inhibitors with potent statins yields a more pronounced effect than the use of either drug in isolation, particularly with evolocumab paired with atorvastatin or rosuvastatin. Meanwhile, there were fewer adverse events reactions in our analysis, but long-term efficacy and safety remained unclear. Thus, further randomized, large-sample, double-blind, and placebo-controlled trials are essential to evaluate the impact of different medicine options on the progression of hyperlipidemia. In future studies, we might investigate the comparative effectiveness of different PCSK9 inhibitors when combined with lipid-lowering treatments such as ezetimibe, bempedoic acid, and other high-efficacy statins through more comprehensive and rigorous network meta-analysis. In conclusion, it is crucial to optimize the effectiveness and safety of lipid-lowering medications in treating metabolic disorders and acknowledge the constraints of indirect comparison in the network meta-analysis.

## Data Availability

The datasets presented in this study can be found in online repositories. The names of the repository/repositories and accession number(s) can be found in the article/Supplementary Material.
