# Supplementary material for: Efficacy and safety of PCSK9 inhibitors, potent statins, and their combinations for reducing low-density lipoprotein cholesterol in hyperlipidemia patients: a systematic network meta-analysis
Source: Front Cardiovasc Med. 2025 Feb 5;11:1415668. doi: 10.3389/fcvm.2024.1415668 (PMC11836037; doi:10.3389/fcvm.2024.1415668)

**Supplementary materials**

**Table S1.** The search strategies of various databases

**Table S2.** Demographic characteristics and clinical manifestations in hyperlipidemia patients

**Table S3.** Node-splitting approach for inconsistency assessment of all comparisons.

**Table S4.** Pooled mean difference, odds ratio and heterogeneity for each direct comparison.

**Table S1**  **Search strategies**

**Pubmed:**

1 "Hyperlipidemias"[Mesh]

2 (((((hyperlipemia[Title/Abstract]) OR (hyperlipidemia[Title/Abstract])) OR (hyperlipoidemia[Title/Abstract])) OR (hyperlipidaemia[Title/Abstract])) OR (Lipidemia[Title/Abstract])) OR (Lipidemias[Title/Abstract])

3 ("Hyperlipidemias"[Mesh]) OR ((((((hyperlipemia[Title/Abstract]) OR (hyperlipidemia[Title/Abstract])) OR (hyperlipoidemia[Title/Abstract])) OR (hyperlipidaemia[Title/Abstract])) OR (Lipidemia[Title/Abstract])) OR (Lipidemias[Title/Abstract]))

4 ((((alirocumab[Title/Abstract]) OR (SAR236553[Title/Abstract])) OR (SAR-236553[Title/Abstract])) OR (REGN-727[Title/Abstract])) OR (monoclonal antibody REGN727[Title/Abstract])

5 (((evolocumab[Title/Abstract]) OR (repatha[Title/Abstract])) OR (AMG-145[Title/Abstract])) OR (AMG 145[Title/Abstract])

6 (((inclisiran[Title/Abstract]) OR (leqvio[Title/Abstract])) OR (ALN-PCSsc[Title/Abstract])) OR (ALN-PCS[Title/Abstract])

7 (((atorvastatin[Mesh]) OR (atorvastatin therapy[Title/Abstract])) OR ((3R,5R)-7-(2-(4-Fluorophenyl)-5-isopropyl-3-phenyl-4-(phenylcarbamoyl)-1H-pyrrol-1-yl)-3,5-dihydroxyheptanoic acid[Title/Abstract])) OR (CI-981[Title/Abstract])

8 (((("Rosuvastatin Calcium"[Mesh]) OR (rosuvastatin[Title/Abstract])) OR (Crestor[Title/Abstract])) OR (ZD4522[Title/Abstract])) OR (ZD 4522[Title/Abstract])

9 #4 or #5 or #6 or #7 or #8

10 #3 and #9 （2876）

**Cochrane Library**

1. MeSH descriptor: [Hyperlipidemias] explode all trees

2 (hyperlipemia OR hyperlipidemia OR hyperlipoidemia OR hyperlipidaemia OR Lipidemia OR Lipidemias):ti,ab,kw

3 #1 or #2

4 (alirocumab OR SAR236553 OR SAR-236553 OR REGN-727 OR monoclonal antibody REGN727):ti,ab,kw

5 (evolocumab OR repatha OR AMG-145 OR AMG 145):ti,ab,kw

6 (inclisiran OR leqvio OR ALN-PCSsc OR ALN-PCS):ti,ab,kw

7 MeSH descriptor: [Atorvastatin] explode all trees

8 (atorvastatin therapy or CI 981 or CI-981 or Lipitor or Liptonorm):ti,ab,kw

9 #7 or #8

10 MeSH descriptor: [Rosuvastatin Calcium] explode all trees

11 (rosuvastatin OR Crestor OR ZD4522 OR ZD 4522):ti,ab,kw

12 #10 or #11

13 #4 or #5 or #6 or #9 or #12

14 #3 and #13 (1597)

**Embase**

1. 'hyperlipidemia'/exp
2. hyperlipemia:ab,ti OR hyperlipidemias:ab,ti OR hyperlipaemia:ab,ti OR hyperlipidaemia:ab,ti OR lipidemia:ab,ti
3. #1 OR #2
4. 'alirocumab'/exp
5. praluent:ab,ti OR sar236553:ab,ti OR 'regn 727':ab,ti OR 'monoclonal antibody regn727':ab,ti
6. #4 OR #5
7. 'evolocumab'/exp
8. 'sal 003':ab,ti OR sal003:ab,ti OR repatha:ab,ti OR 'amg 145':ab,ti
9. #7 OR #8
10. 'inclisiran'/exp
11. 'aln 60212':ab,ti OR aln60212:ab,ti OR 'inclisiran sodium':ab,ti OR 'kjx 839':ab,ti OR leqvio:ab,ti OR 'aln pcssc':ab,ti OR 'aln pcs':ab,ti
12. #10 OR #11
13. 'atorvastatin'/exp
14. ('atorvastatin therapy':ab,ti OR '7 [2 (4 fluorophenyl) 3 phenyl 4 (phenylaminocarbonyl) 5 (2 propanyl) 1 pyrrolyl] 3, 5 dihydroxyheptanoic acid':ab,ti OR 'ci 981':ab,ti OR '3 phenyl 4 phenylcarbamoyl 1h pyrrole 1 heptanoic acid':ab,ti OR 'a 2581175':ab,ti
15. #13 OR #14
16. 'rosuvastatin'/exp
17. 'rosuvastatin calcium':ab,ti OR crestor:ab,ti OR zd4522:ab,ti OR 'zd 4522':ab,ti
18. #16 OR #17
19. #6 OR #9 OR #12 OR #15 OR #18
20. #3 AND #19 (14870)

**TABLE S2** | **Characteristics of included studies**

| **Study name** | **Population** | **Sex（male/female）** | | **Age(years)** | | **Duration** | **Intervention** |
| --- | --- | --- | --- | --- | --- | --- | --- |
|  |  | Text group | Control group | Text group | Control  group |  |  |
| Blom (2019) | HeFH | 41/64 | 28/34 | 55.6 (12.6) | 55.4 (10.7) | 144w | Alirocumab 75mg/150mg Q2W(n=105) VS placebo(n=62) |
| Colhoun (2014) | hypercholesterolemia | 131/78 | 77/30 | 63.0(9.5) | 63.0(8.8) | 52w | Alirocumab 75mg/150mg Q2W(n=205) VS placebo(n=106) |
| Chao (2019) | hypercholesterolemia | 29/28 | 27/32 | 61.5 (11.1) | 60.0 (8.9) | 24w | Alirocumab 75mg/150mg Q2W(n=57) VS placebo(n=59) |
| Koren (2015) | hypercholesterolemia | 47/61 | 38/39 | 58.2 (10.1) | 53.8 (9.4) | 12w | Alirocumab 150mg Q2W (n=108) VS placebo(n=77) |
| Jennifer (2014)  QDYSSEYⅠ(NCT01730040) | hypercholesterolemia | 31/16 | 33/14 | 64.2 (10.36) | 63.2 (10.89) | 24w | Alirocumab 75mg/150mg Q2W + atorvastatin 40mg(n=46) VS atorvastatin 80mg (n=47) |
| Jennifer (2014)  OPTIONSⅡ(NCT01730053) | hypercholesterolemia | 31/18 | 33/15 | 62.2 (11.11) | 61.5 (11.15) | 24w | Alirocumab 75mg/150mg Q2W + Rosuvastatin 10mg (n=48) VS Rosuvastatin 20mg (n=48) |
| Toth (2016) | hypercholesterolemia | 13/16 | 13/13 | 58.2 (10.1) | 53.8 (9.4) | 8w | Alirocumab 150mg Q2W +atorvastatin 80mg (n=29) VS placebo + atorvastatin 80mg (n=26) |
| Teramoto (2017) | hypercholesterolemia | 33/20 | 37/19 | 63.6 (10.4) | 64.6 (10.0) | 12W | Alirocumab 150mg Q2W +atorvastatin 5mg (n=53) VS placebo + atorvastatin 5mg (n=56) |
| Nam (2018) | hypercholesterolemia | 32/8 | 35/8 | 60.8 (9.4) | 60.2 (9.4) | 24w | Alirocumab 75/150mg Q2W (n=40) VS placebo (n=43) |
| Teramoto (2016) | hypercholesterolemia | 9/16 | 14/11 | 58.2 (8.8) | 58.6 (9.2) | 12w | Alirocumab 150mg Q2W (n=25) VS placebo (n=25) |
| Teramoto (2016) | HeFH | 84/60 | 47/25 | 60.3±9.7 | 61.8±9.0 | 24w | Alirocumab 75/150mg Q2W (n=144) VS placebo (n=72) |
| Farnier (2016) | hypercholesterolemia | 397/302 | 216/136 | 55.6 (12.9) | 55.5 (12.5) | 24w | Alirocumab 75/150mg Q2W (n=693) VS placebo (n=350) |
| Moriarty (2016) | HeFH | 26/15 | 10/11 | 59.5 (9.2) | 57 (10.5) | 6w | Alirocumab 150mg Q2W (n=41) VS placebo (n=21) |
| Kastelein (2017) | HeFH | 266/224 | 139/106 | 52.5 (12.9) | 52.2 (12.4) | 24w | Alirocumab 75/150mg Q2W (n=488) VS placebo (n=244) |
| Roth (2020) | hypercholesterolemia | 66/80 | 40/33 | 59.2 (10.8) | 59.4 (10.2) | 24w | Alirocumab 300mg Q4W /150mg Q2W (n=144) VS placebo (n=71) |
| Kereiakes (2015) | hypercholesterolemia | 131/78 | 77/30 | 63.0 (9.5) | 63.0 (8.8) | 24w | Alirocumab 75mg/150mg Q2W (n=205) VS placebo (n=106) |
| Ginsberg (2016) | HeFH | 35/37 | 22/13 | 49.8 (14.2) | 52.1 (11.2) | 24w | Alirocumab 150mg Q2W (n=71) VS placebo (n=35) |
| Koren (2015) | hypercholesterolemia | 13/16 | 16/15 | 59.9 (10.7) | 53.3 (8.5) | 12w | Alirocumab 150mg Q2W (n=29) VS placebo (n=31) |
| Stroes (2016) | hypercholesterolemia | 69/47 | 31/27 | 62.5 (9.9) | 63.1 (10.7) | 24w | Alirocumab 75mg/150mg Q2W (n=115) VS placebo (n=57) |
| Blom (2020) | HoFH | 21/24 | 13/11 | 42.3(14.1) | 45.4 (15.8) | 12w | Alirocumab 150mg Q2W (n=45) VS placebo (n=24) |
| Kastelein (2015) | HeFH | 180/143 | 94/69 | 52.1 (12.9) | 51.7+12.3 | 24w | Alirocumab 75mg/150mg Q2W (n=322) VS placebo (n=163) |
| Robinson (2015) | hypercholesterolemia | 983/570 | 474/314 | 60.4 (10.4) | 60.6 (10.4) | 24w | Alirocumab 150mg Q2W (n=1530) VS placebo (n=780) |
| Roth (2016) | hypercholesterolemia | 190/122 | 101/56 | 61.6 (10.0) | 61.6 (9.7) | 24w | Alirocumab 300mg Q4W (n=308) VS placebo (n=156) |
| Fariner (2018) | HeFH | 267/208 | 130/106 | 54.1 (12.1) | 54.8 (11.4) | 96w | Alirocumab 75mg/150mg Q2W (n=475) VS placebo (n=236) |
| Lorenzatti (2018) | T2DM and hyperlipidemia | 149/178 | 64/102 | 61.0 (8.5) | 61.6 (8.8) | 12w | Evolocumab 140mg Q2W+atorvastatin 20mg (n=325) VS placebo + atorvastatin (n=164) |
| Kiyosue (2015) | Hyperlipidemia  Mixed dyslipidemia | 31/20 | 29/19 | 62.0 (11) | 61.0 (10) | 12w | Evolocumab 140mg Q2W+atorvastatin 20mg (n=51) VS placebo + atorvastatin (n=48) |
| Toth (2017) | hyperlipidemia | 170/166 | 126/157 | 56.4（10.6） | 57.1（10.0） | 52w | Evolocumab 420mg Q4W (n=309) VS placebo (n=250) |
| Hirayama (2014) | Hypercholesterolemia | 32/20 | 36/16 | 60.8 (9.2) | 60.2 (10.1) | 12w | Evolocumab 140mg Q2W (n=52) VS placebo (n=52) |
| Gaudet (2018) | HeFH | 43/61 | 26/27 | 13.7 (2.3) | 13.7 (2.5) | 24w | Evolocumab 420mg Q4W (n=104) VS placebo (n=53) |
| Rosenson (2016) | Mixed hyperlipidemia | - | - | - | - | 12w | Evolocumab 140mg Q2W /420mg Q4W (n=1167) VS placebo (n=546) |
| Blom (2017) | Mets  Hypercholesterolemia | 290/309 | 141/161 | 55.9 (10.9) | 56.6 (10.3) | 52w | Evolocumab 420mg Q4W +atorvastatin 80mg (n=599) VS placebo+atorvastatin 80mg (n=302) |
| Chen (2019) | Dyslipidemia  hyperlipidemia | 77/75 | 34/40 | 61 (11.5) | 60 (11) | 12w | Evolocumab 420mg Q4W +atorvastatin 20mg (n=152) VS placebo+atorvastatin 20mg (n=74) |
| Koren (2014) | Hypercholesterolemia | 52/101 | 13/65 | 53 (12) | 53 (11) | 12w | Evolocumab 420mg Q4W (n=153) VS placebo (n=78) |
| Desai (2013) | Hypercholesterolemia | 33/75 | 36/42 | 62.4 (10.2) | 60.2 (8.8) | 12w | Evolocumab 140 mg Q2W (n=78) VS placebo (n=78) |
| Raal (2012) | HeFH | 35/21 | 24/32 | 51.8 (13.0) | 49.3 (11.3) | 12w | Evolocumab 420mg Q4W (n=56) VS placebo (n=56) |
| Santos (2022) | HeFH | 42/59 | 25/24 | 15·0 (1.0) | 14·0 (1.0) | 12w | Evolocumab 420mg Q4W (n=101) VS placebo (n=49) |
| Robinson (2014)  (NCT01763866) | Hypercholesterolemia | 66/44 | 27/28 | 59.6 (9.9) | 59.9 (10.2) | 12w | Evolocumab 420mg Q4W +atorvastatin 80mg (n=110) VS placebo (n=55) |
| Robinson (2014)  (NCT01588496) | Hypercholesterolemia | 63/49 | 29/26 | 59.6 (9.9) | 59.9 (10.2) | 12w | Evolocumab 420mg Q4W +Rosuvastatin 40mg (n=112) VS placebo (n=55) |
| Raal (2015) | HoFH | 17/16 | 8/8 | 30 (12) | 32 (14) | 12w | Evolocumab 420mg Q4W (n=33) VS placebo (n=16) |
| Raal (2015) | HeFH | 66/44 | 29/25 | 52·6 (12·3) | 51·1 (14·2) | 12w | Evolocumab 140mg Q4W (n=110) VS placebo (n=54) |
| Santos (2020) | HeFH | 43/61 | 26/27 | 13.7(2.3) | 13.7(2.5) | 24w | Evolocumab 420mg Q4W (n=104) VS placebo (n=53) |
| Blom (2014) | hyperlipidemia | 109/145 | 59/70 | 57.2(10.3) | 57.0(10.6) | 52w | Evolocumab 420mg Q4W +atorvastatin 10mg (n=254) VS placebo+atorvastatin (n=129) |
| Raal (2022) | HeFH  ASCVD | 87/61 | 81/69 | 58.3 (10.3) | 58.9 (11.5) | 510days | Inclisiran sodium 300mg (n=130) VS placebo (n=137) |
| Ray (2019) | ASCVD  hypercholesterolemia  diabetes | 45/16 | 33/29 | 64.1 (9.4) | 62.8 (10.3) | 24w | Inclisiran sodium 300mg 1+90 days (n=59) VS placebo (n=61) |
| Wright (2020) | HeFH  ASCVD | 1226/607 | 1244/583 | 64.1（9.98） | 63.9（9.87） | 510days | Inclisiran sodium 300mg (n=1646) VS placebo (n=1634) |
| Ray (2022) | ASCVD  Elevated cholesterol | 45/53 | 50/55 | 62.7 (10.6) | 63.6 (9.2) | 510 days | Inclisiran 284mg (n=98) VS placebo (n=105) |
| Fitzgerald (2014) | Healthy volunteers with raised cholesterol | 22/2 | 8/0 | 51.0（8.75） | 41.5（7.0） | 30 days | Inclisiran 400mg/kg (n=6) VS placebo (n=8) |
| Fitzgerald (2016) | hypercholesterolemia | 17/1 | 2/4 | 46.0（10） | 48.0（14） | 84days | Inclisiran 300mg (n=3) VS placebo (n=6) |
| Raal (2020) | HeFH | 112/130 | 115/125 | 56 .0 (4.0) | 56.0 (46–64) | 510days | Inclisiran 300mg (n=242) VS placebo (n=240) |
| Ray (2020) | ASCVD  Elevated cholesterol | 535/246 | 548/232 | 66.4（8.9） | 65.7（8.9） | 510days | Inclisiran 284mg (n=781) VS placebo (n=780) |
| Luo (2023) | hypercholesterolemia | 3/12 | 4/6 | 59.5（7.45） | 57.3（9.59） | 90days | Inclisiran 300mg (n=15) VS placebo (n=10) |
| Mccrindle (2003) | Familial hypercholesterolemia | 95/45 | 34/13 | 14.1（2.0） | 14.1（2.2） | 26w | Atorvastatin 10-20mg (n=140) VS placebo (n=47) |
| Hernandez (2011) | hypercholesterolemia | 6/35 | 3/13 | 58.73 (7.91) | 59.06（8.82） | 12w | Atorvastatin 10mg (n=22) VS placebo (n=21) |
| Bakker-arkema (2013) | Hypertriglyceridemia | 12/4 | 12/2 | 52.0（6.75） | 49.5（10.25） | 4w | Atorvastatin 20mg (n=16) VS placebo (n=14) |
| Wang (2011) | Elevated LDL-cholesterol | 14/12 | 18/10 | 66.8（8.6） | 65.4（9.0） | 8w | Atorvastatin 10mg (n=26) VS placebo (n=28) |
| Tanaka (2001) | T2DM  hypercholesterolemia | 12/7 | 9/9 | 61（5） | 55（11） | 12w | Atorvastatin 10mg (n=18) VS placebo (n=18) |
| Heinonen (1996) | Primary hypercholesterolemia | 10/8 | 11/6 | 51（2.0） | 56（2.7） | 26w | Atorvastatin 10mg (n=18) VS placebo (n=17) |
| Bregar (2009) | hypercholesterolemia | - | - | 61.3（2.3） | 60.1（1.4） | 12w | Atorvastatin 20mg (n=17) VS placebo (n=20) |
| Schrott (2000) | Overweight patients | - | - | 47 | 47 | 4w | Atorvastatin 10mg (n=11) VS placebo (n=11) |
| Hamilton (2010) | hyperlipidaemia | 10/10 | 11/9 | 49.7 (7.5) | 50.7 (8.0) | 8w | Atorvastatin 10-20mg (n=20) VS placebo (n=20) |
| Vansant (2001) | Postprandial lipaemia  obesity | - | - | 47.3（8.6） | 32.7（8.8） | 8w | Atorvastatin 20mg (n=15) VS placebo (n=7) |
| Laffin (2023) | hyperlipidemia | 11/14 | 14/11 | 62.7（6.0） | 63.5（7.1） | 4w | Rosuvastatin 5mg (n=24) VS placebo (n=23) |
| Kennedy (2011) | hyperlipidemia | 17/0 | 15/0 | 64.5（9.33） | 64.5（9.33） | 8w | Rosuvastatin 5-10mg (n=15) VS placebo (n=17) |
| Avis (2010) | Familial hypercholesterolemia | 22/23 | 24/22 | 14.5 (1.8) | 14.5 (1.8) | 12w | Rosuvastatin 20mg (n=44) VS placebo (n=46) |
| Hunninghake (2004) | Hypertriglyceridemia | 15/8 | 14/12 | 58.2 (8.8) | 55.6 (11.9) | 6w | Rosuvastatin 10mg (n=21) VS placebo (n=24) |
| Caslake (2003) | hyperlipidaemia | 11/5 | 11/5 | - | - | 8w | Rosuvastatin 40mg (n=16) VS placebo (n=16) |
| Shepherd (2004) | hypercholesterolemia | - | - | 57.4 (8.1) | 59.2 (8.1) | 6w | Rosuvastatin 10mg (n=44) VS placebo (n=46) |
| Olsson (2001) | hypercholesterolemia | - | - | 56 | 56 | 6w | Rosuvastatin 20mg (n=13) VS placebo (n=29) |
| Saito (2003) | hypercholesterolemia | 10/9 | 7/8 | 56.4 (12.0) | 58.5 (12.6) | 6w | Rosuvastatin 20mg (n=18) VS placebo (n=12) |
| Talavera (2013) | Hypertriglyceridemia | 45/67 | 57/54 | 53.14 (12) | 51.48 (13.47) | 8w | Rosuvastatin 20mg (n=111) VS placebo (n=111) |

**Table S3. Node-splitting approach for inconsistency assessment of all comparisons**

**LDL-C**

| **Side** | **Direct**  **Coef. Std. Err** | **Indirect**  **Coef. Std. Err** | **Difference**  **Coef. Std. Err** | **P>\|z\|** |
| --- | --- | --- | --- | --- |
| A C -1.689017 0.194943 -1.521331 0.6376161 -0.1676864 0.6666726 0.801  A E -1.911406 0.2001589 -2.078935 0.6359981 0.1675286 0.6666727 0.802  C D * -1.3 0.6122058 -0.9643665 1.185015 -0.3356333 1.333271 0.801  C E * -0.3899987 0.6053902 -0.2223886 0.2792167 -0.16761 0.6666778 0.801  C F * -1.31 0.5977714 -0.9746272 1.177708 -0.3353728 1.333346 0.801  D E * 0.9099969 0.6059863 1.244717 1.194563 -0.3347204 1.333269 0.802  D F * -0.01 0 .5934504 -5.313518 177.6443 5.303518 177.6454 0.976  D G * 1.210744 0.4150617 4.06291 96.56619 -2.852166 96.56708 0.976  E F * -0.92 0.5914005 -1.255057 1.187317 0.3350574 1.333345 0.802  G H * -1.712155 0.2128181 3.765042 200.2261 -5.477197 200.2262 0.978  G J * -1.74 0.5747518 -1.768263 1.089669 0.0282635 1.241552 0.982  G K * -0.09 0.5926192 -0.1182635 1.099197 0.0282635 1.241552 0.982  H J * -0.04 0.5654167 -0.0117079 1.104223 -0.0282921 1.241552 0.982  H K * 1.61 0.5835701 1.638292 1.113628 -0.0282921 1.241553 0.982  J K * 1.649993 0.529751 -2.450877 0.5297512 4.10087 0 NA | | | | |

**AEs**

| **Side** | **Direct**  **Coef. Std. Err** | **Indirect**  **Coef. Std. Err** | **Difference**  **Coef. Std. Err** | **P>\|z\|** |
| --- | --- | --- | --- | --- |
| A C .228546 .2979607 1.449762 .5230571 -1.221216 .6018774 0.042  A E .2796932 .2805175 -.9415242 .5326152 1.221217 .6018773 0.042  C D * -.8283219 .436827 1.614084 1.121363 -2.442406 1.203749 0.042  C E * -1.170071 .4414739 .0511473 .4090939 -1.221219 .6018779 0.042  C F * -1.265381 .4451055 1.177051 1.12462 -2.442432 1.203755 0.042  D E * -.3417492 .441055 2.100665 1.116388 -2.442414 1.20375 0.042  D F * -.4370595 .4471047 .1478401 566.7277 -.5848996 566.7279 0.999  D G * .2231436 .4604832 -.0924906 313.8856 .3156342 313.8859 0.999  E F * -.0953102 .4492556 -2.537745 1.119658 2.442435 1.203755 0.042  G H * .1078592 .1261688 -.5146616 616.3019 .6225208 616.302 0.999  G J * -.1436427 .3569682 .4525523 .6184977 -.596195 .7645465 0.436  G K * 2.43e-11 .4058437 .596195 .6479366 -.596195 .7645462 0.436  H J * .010508 .3015862 -.5856875 .701394 .5961955 .7645455 0.436  H K * .1541507 .3581022 -.4420456 .7274852 .5961963 .7645444 0.436 | | | | |

**Note:** A:Placebo; B:Alirocumab; C:Atorvastatin; D:Alirocumab+Atorvastatin (Ali+Ator);E: Rosuvastatin; F:Alirocumab+ Rosuvastatin (Ali+Ros); G: Placebo+ Atorvastatin (Placebo+ Ator); H: Evolocumab + atorvastatin (Evo+Ator); I:Evolocumab; J:Evolocumab+Rosuvastatin(Evo+Ros); K:Placebo+ Rosuvastatin(Placebo+ Ros); L: Inclisiran (Inc).

**TABLE S4** | Pooled mean difference, odds ratio and heterogeneity for each direct comparison in LDL reducing and adverse events reaction

| **Comparison** | **Number of RCTs** | **Number of participants** | **(Pooled) MD (95% CI)** | **I square (%)** | **P value** |
| --- | --- | --- | --- | --- | --- |
| **LDL-C** | | | | | |
| Ali vs Placebo | 20 | 7868 | -1.82 (-1.99, -1.64) | 90.5 | < 0.001 |
| Evo vs Placebo | 10 | 3396 | -1.91 (-2.24, -1.59) | 93.2 | < 0.001 |
| Inc vs Placebo | 9 | 5964 | -1.66 (-1.92, -1.40) | 83.4 | < 0.001 |
| Ator vs Placebo | 10 | 506 | -1.68 (-2.11, -1.25) | 92.0 | < 0.001 |
| Ros vs Placebo | 9 | 625 | -1.89 (-2.48, -1.29) | 95.4 | < 0.001 |
| Ali + Ator vs Ator | 1 | 93 | NA | NA | NA |
| Ali+ Ator vs  Placebo +Ator | 2 | 164 | -1.19 (-1.51, -0.87) | 20.9 | 0.261 |
| Evo +Ator vs Placebo +Ator | 7 | 2409 | -1.70 (-1.84, -1.57) | 65 | 0.009 |
| Ali+ Ros vs Ros | 1 | 96 | 0.178(-0.062,0.417) | NA | NA |
| Evo+Ros vs Placebo + Ros | 1 | 167 | NA | NA | NA |

| **AEs OR (95% CI)** | | | | | |
| --- | --- | --- | --- | --- | --- |
| Ali vs Placebo | 19 | 6825 | 1.10 (0.94, 1.28) | 17.8 | 0.236 |
| Evo vs Placebo | 9 | 2837 | 1.09 (0.87, 1.36) | 22.5 | 0.243 |
| Inc vs Placebo | 8 | 5955 | 1.03 (0.92, 1.17) | 0 | 0.510 |
| Ator vs Placebo | 5 | 342 | 1.25 (0.71, 2.19) | 0 | 0.842 |
| Ros vs Placebo | 6 | 294 | 1.33 (0.76, 2.31) | 3.4 | 0.395 |
| Ali + Ator vs Ator | 1 | 93 | NA | NA | NA |
| Ali+ Ator vs  Placebo +Ator | 1 | 109 | NA | NA | NA |
| Evo +Ator vs Placebo +Ator | 5 | 1764 | 1.14 (0.92, 1.41) | 0 | 0.679 |
| Ali+ Ros vs Ros | 1 | 96 | NA | NA | NA |
| Evo+Ros vs Placebo + Ros | 1 | 167 | NA | NA | NA |

| **Neurological AEs in PCSK9i** | | | | | |
| --- | --- | --- | --- | --- | --- |
| Ali vs Placebo | 15 | 4868 | 1.12 (0.85, 1.49) | 0 | 0.947 |
| Evo vs Placebo | 5 | 821 | 2.02 (0.85, 4.79) | 20.1 | 0.287 |
| Inc vs Placebo | 1 | 123 | 0.53 (0.17, 1.67) | 0 | NA |
| Ali + Ator vs Ator | NA | NA | NA | NA | NA |
| Ali+ Ator vs  Placebo +Ator | 1 | 109 | NA | NA | NA |
| Evo +Ator vs Placebo +Ator | 4 | 1764 | 1.02 (0.50, 2.10) | 50.4 | 0.109 |
| Ali+ Ros vs Ros | NA | NA | NA | NA | NA |
| Evo+ Ros vs Placebo + Ros | NA | NA | NA | NA | NA |

| **Neurocognitive AEs in PCSK9i** | | | | | |
| --- | --- | --- | --- | --- | --- |
| Ali vs Placebo | 9 | 4765 | 0.91 (0.47, 1.75) | 0.6 | 0.419 |
| Evo vs Placebo | 1 | 594 | 0.72 (0.12, 4.45) | 0 | NA |
| Inc vs Placebo | NA | NA | NA | NA | NA |
| Ali + Ator vs Ator | 1 | 96 | 0.20 (0.01, 4.19) | 0 | NA |
| Ali+ Ator vs  Placebo +Ator | NA | NA | NA | NA | NA |
| Evo +Ator vs Placebo +Ator | NA | NA | NA | NA | NA |
| Ali+ Ros vs Ros | 1 | 96 | 0.19 (0.01, 4.10) | 0 | NA |
| Evo+ Ros vs Placebo + Ros | NA | NA | NA | NA | NA |

Notes: CI: confidence interval; NA: not applicable; MD: mean difference; OR: Odds ratio


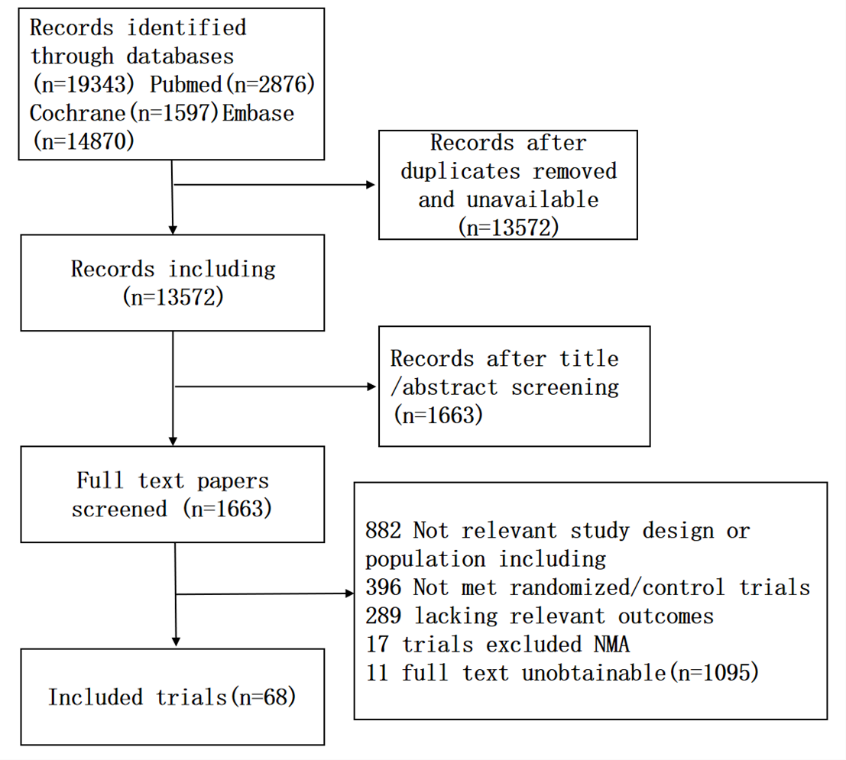
 **Figure 1.** Study flow diagram of the systematic review.

**Figure 2. Risk of Bias Assessment in all included trials**


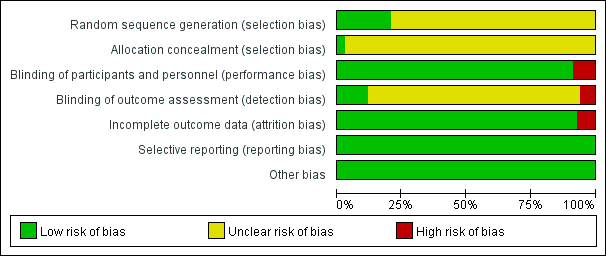


**Figure 3.** The network of eligible treatment comparisons for hyperlipidemia patients


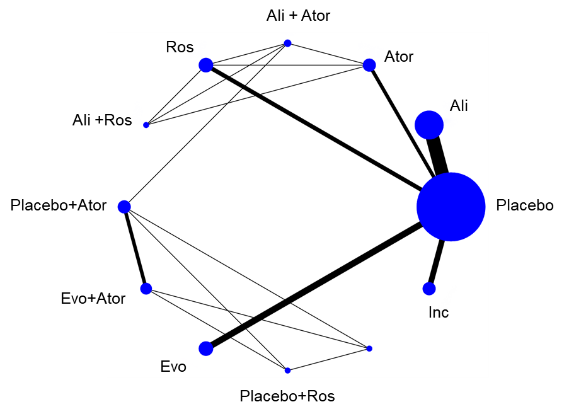

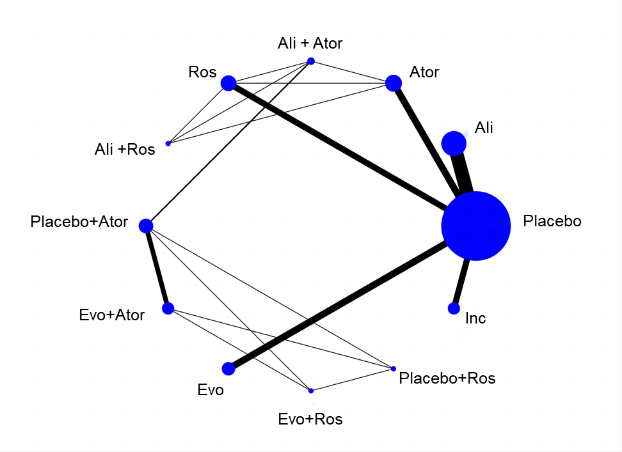


A B

**Figure 4.** The funnel chart in terms of reducing LDL-C (A) and adverse events (B)


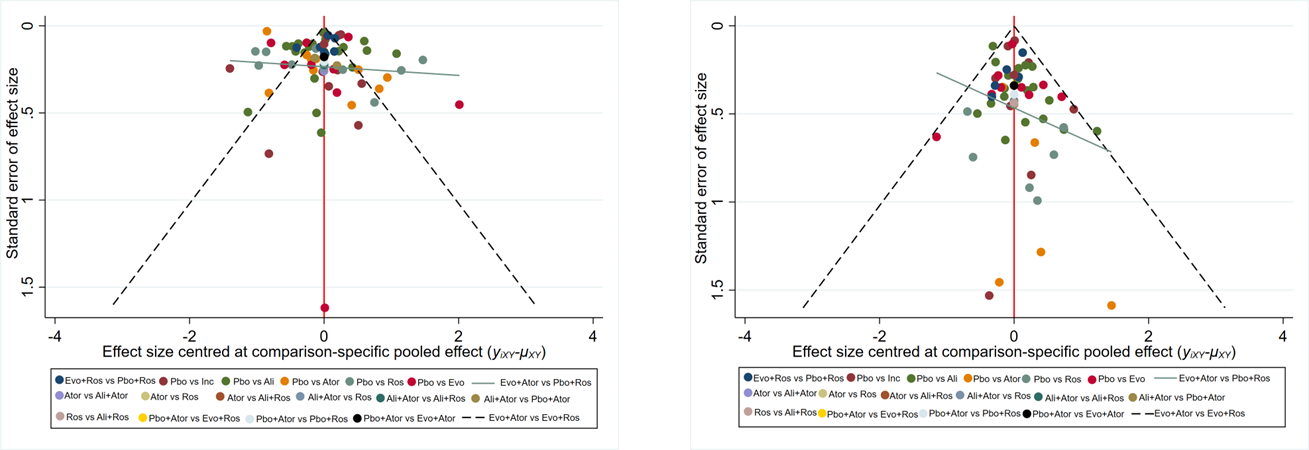


A B

**Figure 5.** Ranking of treatment strategies based on probability of their efficiency


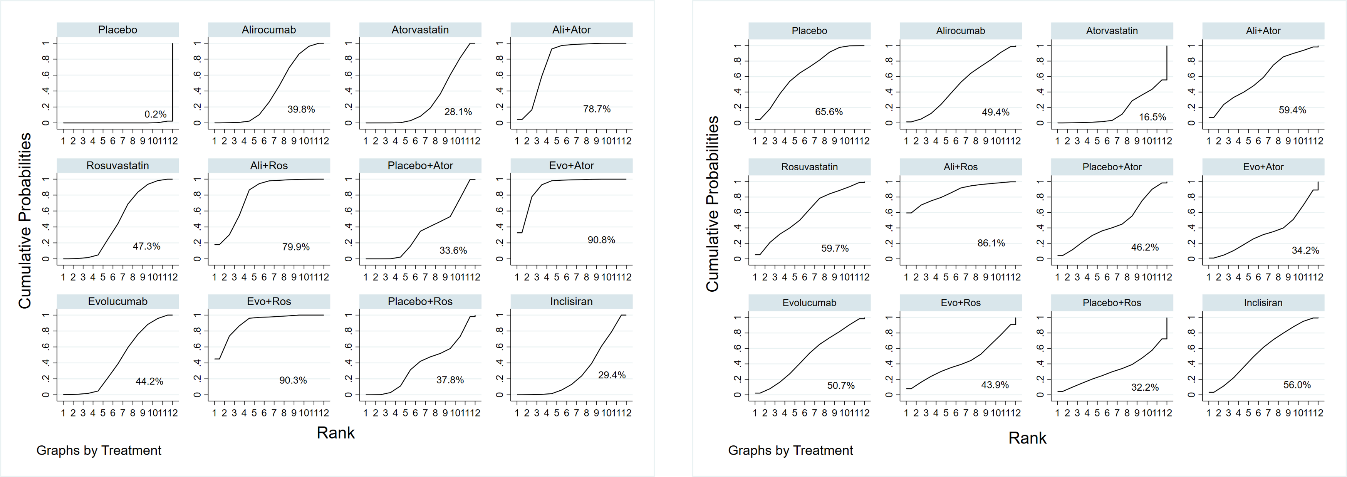


A B

**Figure 6.** Treatment efficiency comparisons for LDL-C lowering (A) and adverse events reactions(B)


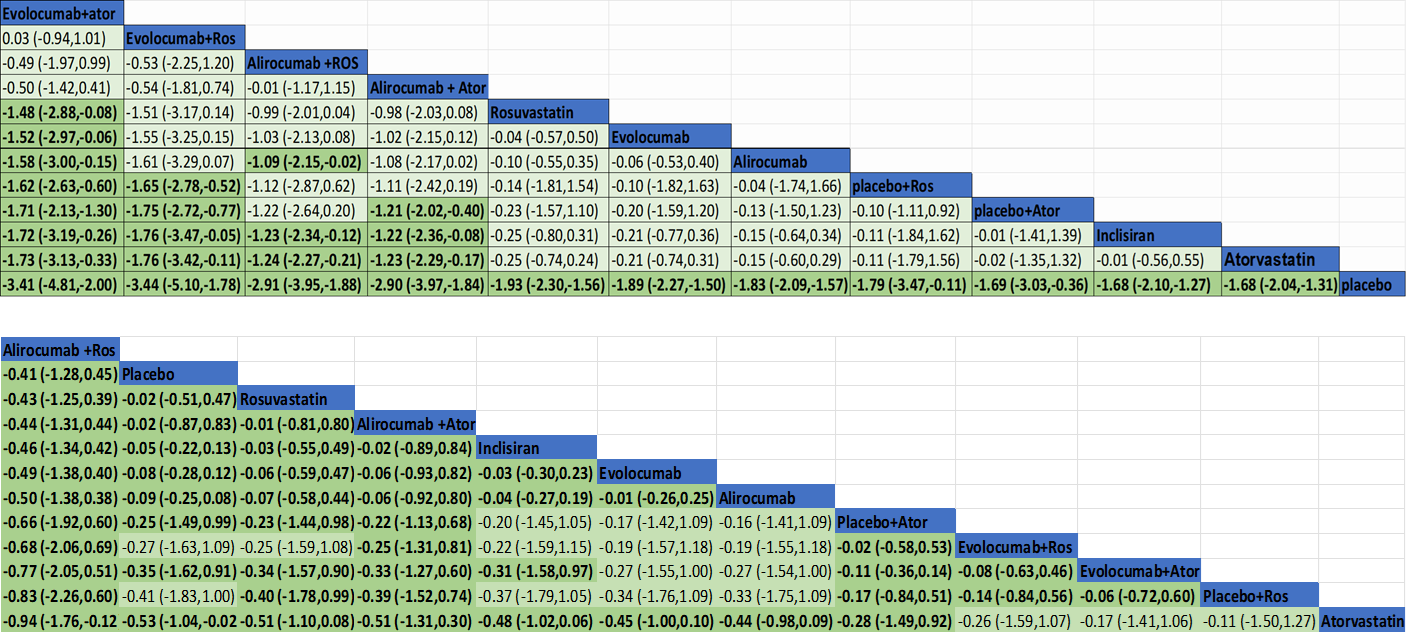


**Figure S1.** The forest plot of overall study consistency in LDL-C reducing (A) and adverse events reactions(B)


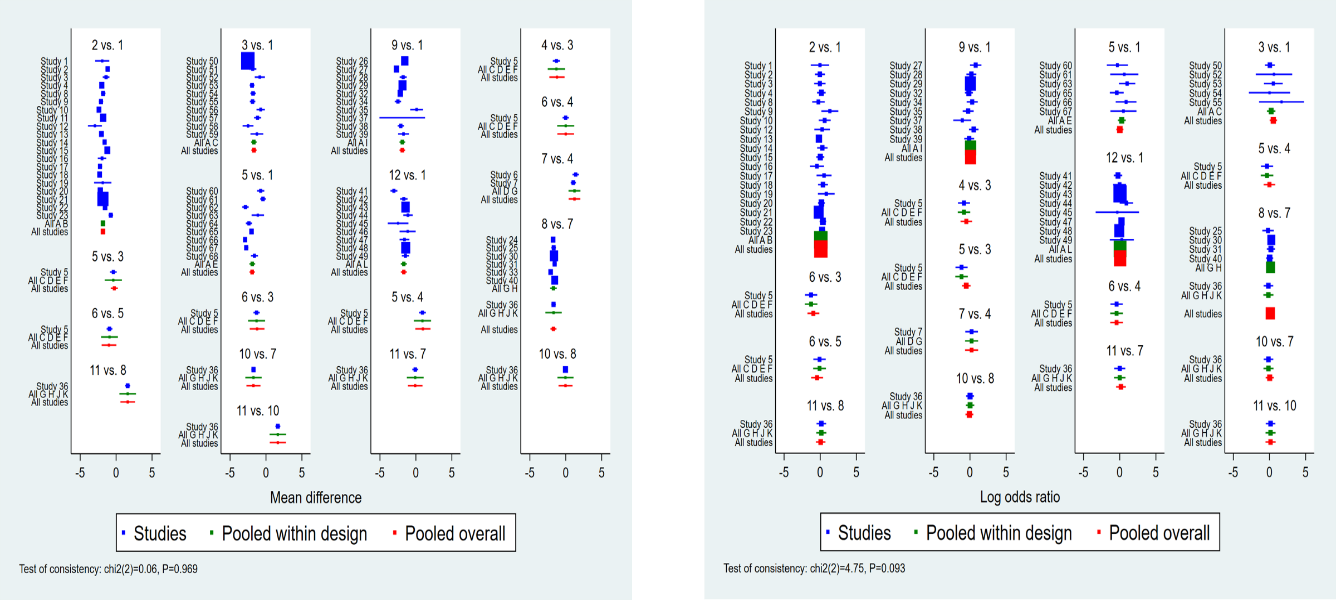


A B

**Figure S2.** The forest plot of direct evidence for LDL-C reducing (mean difference (MD) and 95% confidence interval (CI))


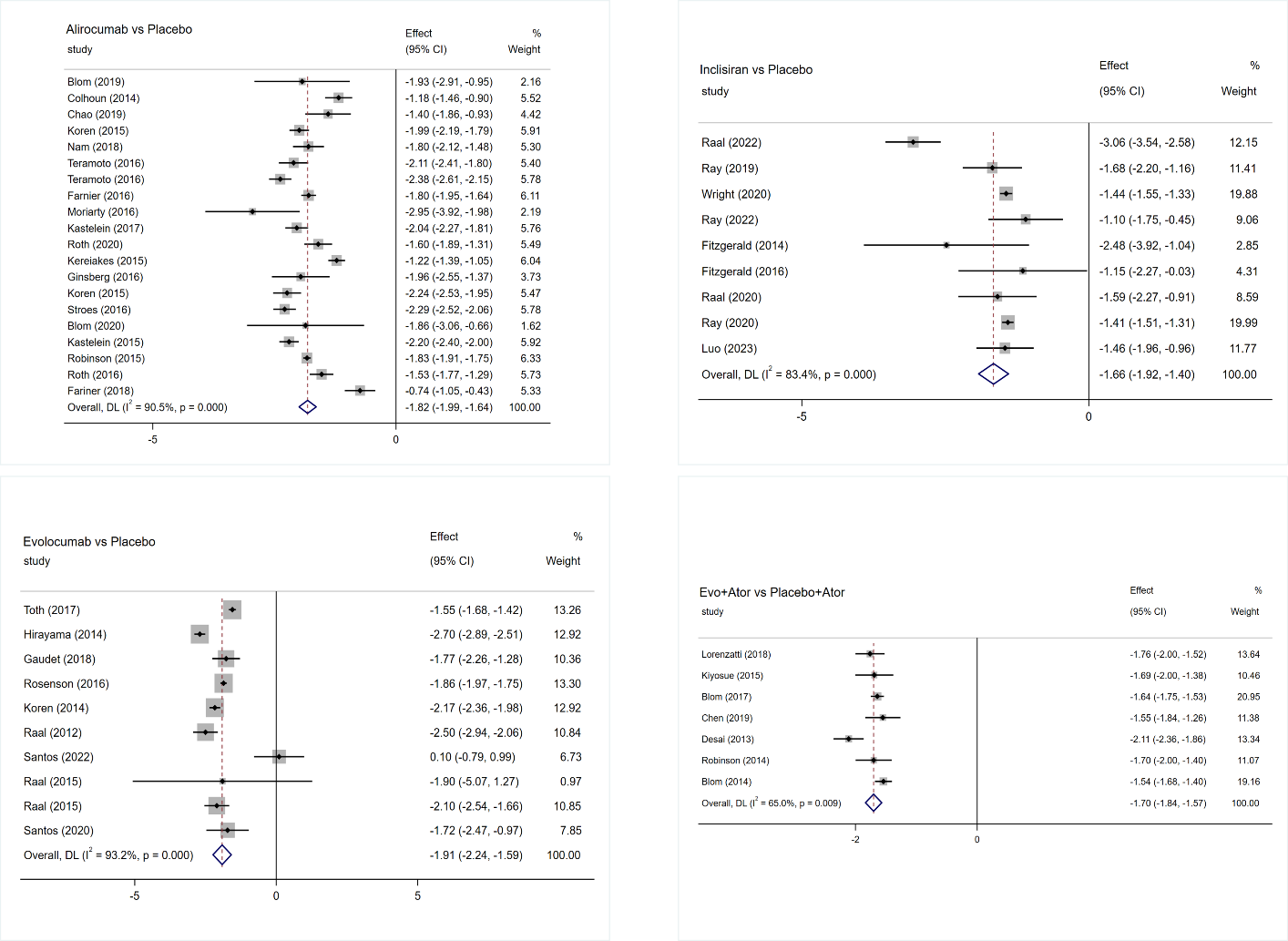


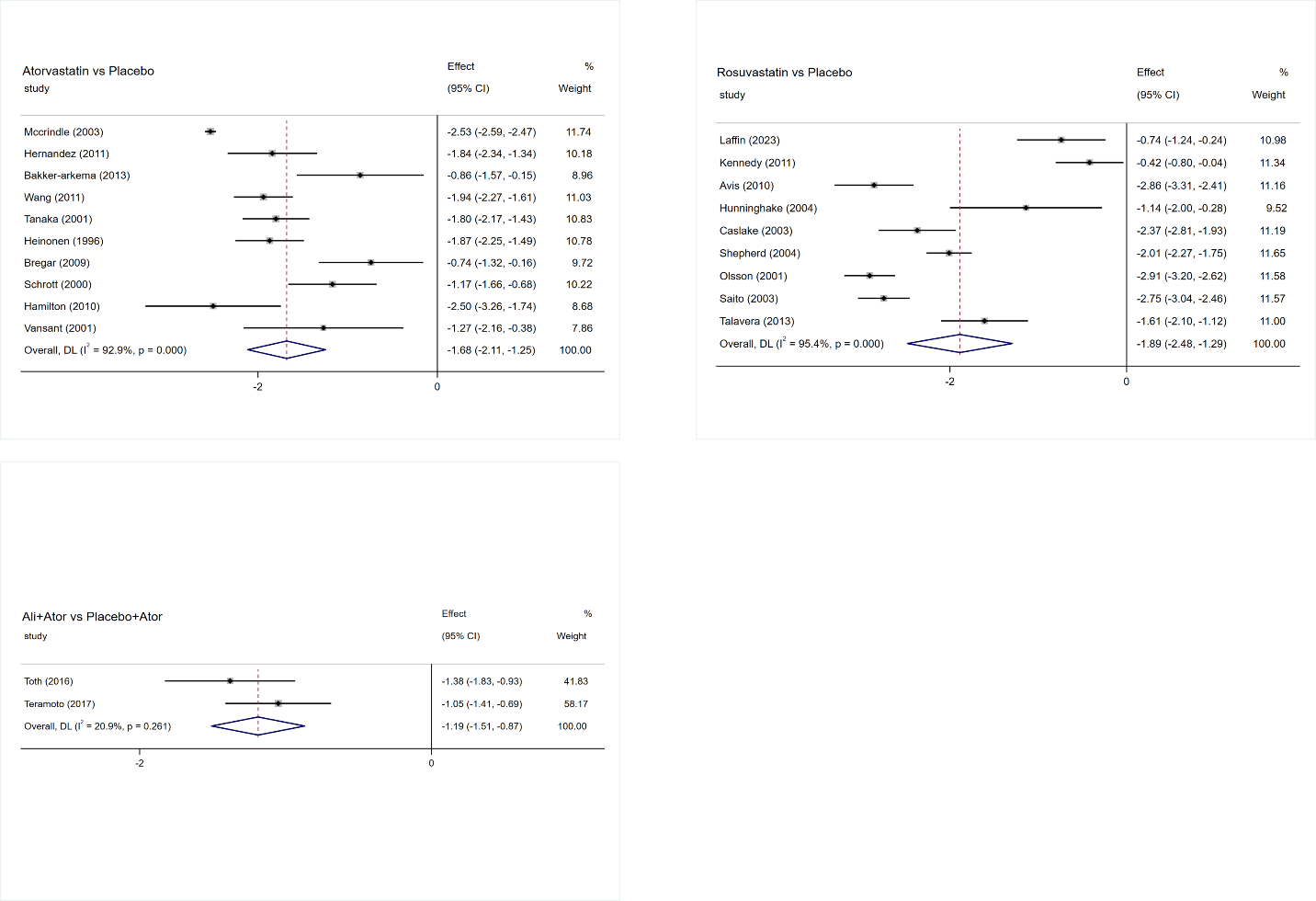


**Figure S3.** The sensitivity analyses for the reducing of LDL-C level


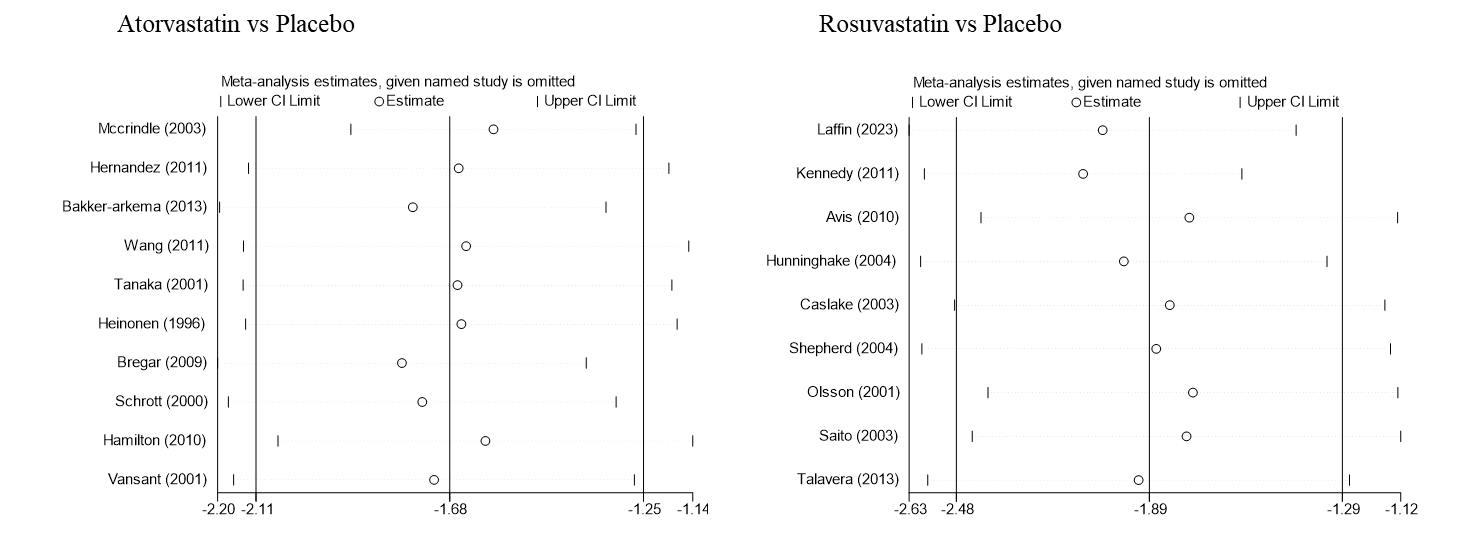

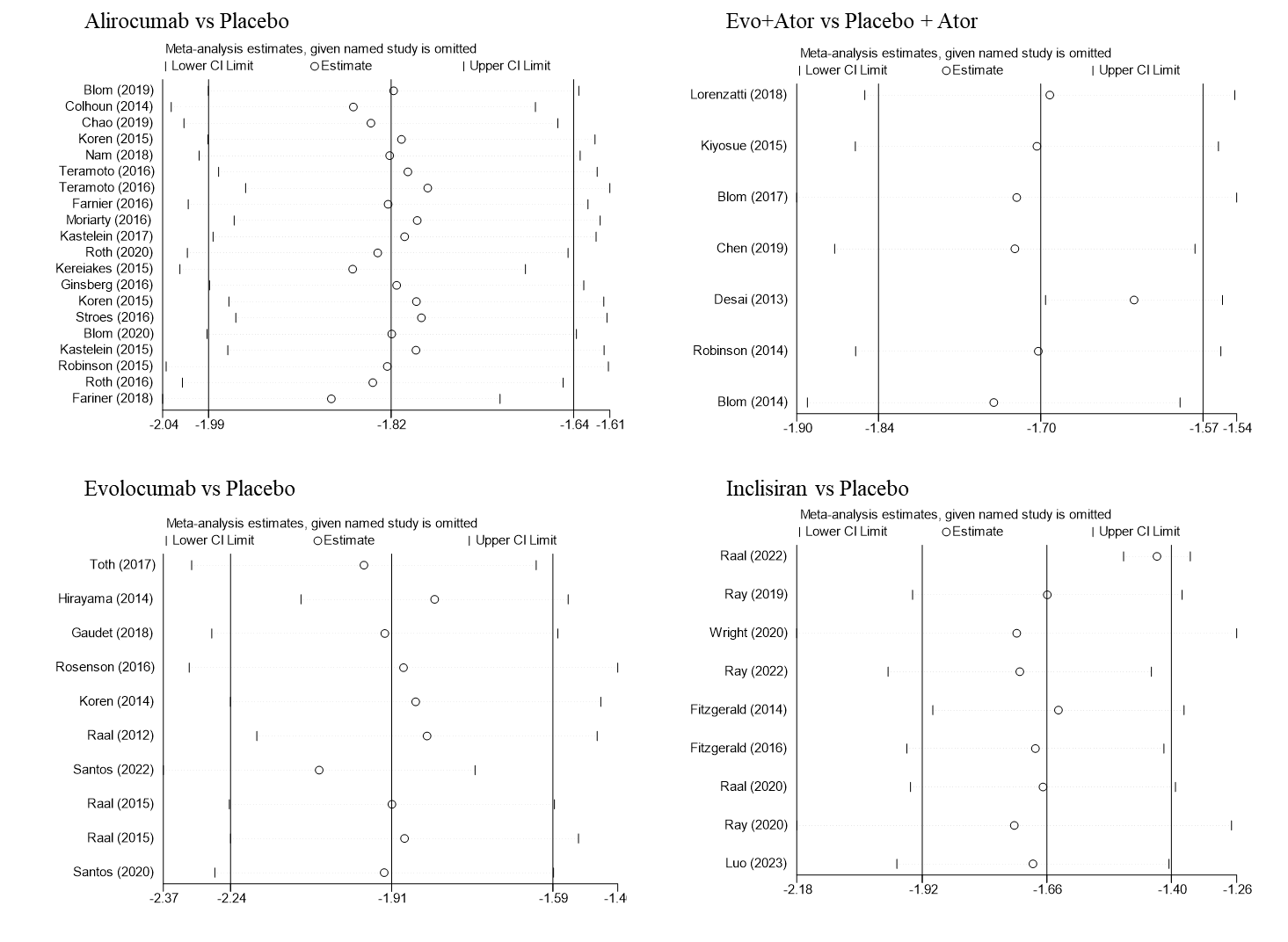


**Figure S4.** The forest plot of direct evidence for AEs incidence (Odds ratio (OR) and 95% confidence interval (CI))


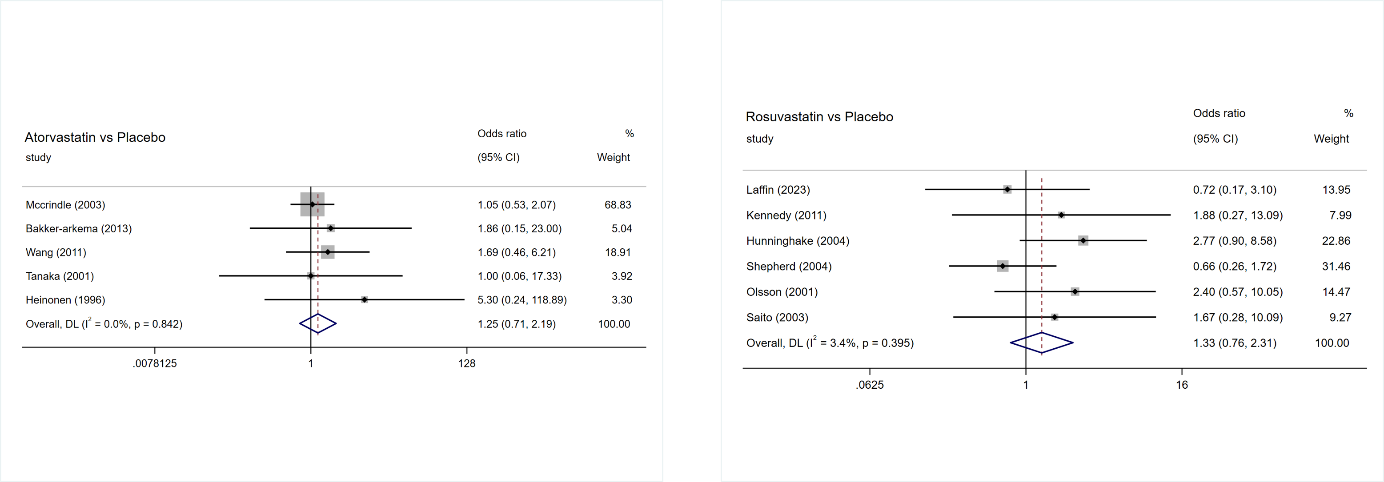

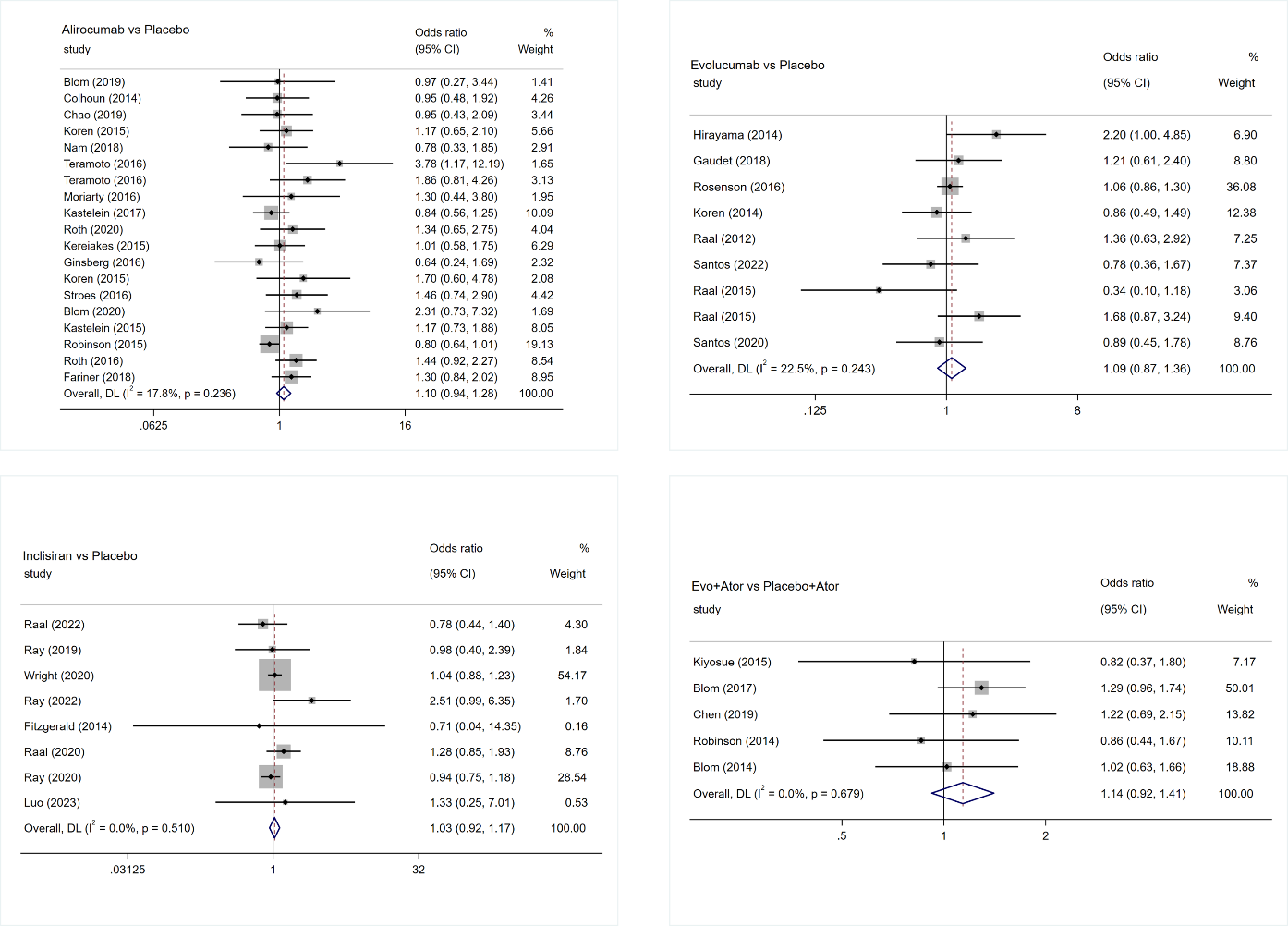


**Figure S5.** The forest plot of direct evidence for the incidence of neurological adverse events (OR and 95% CI )


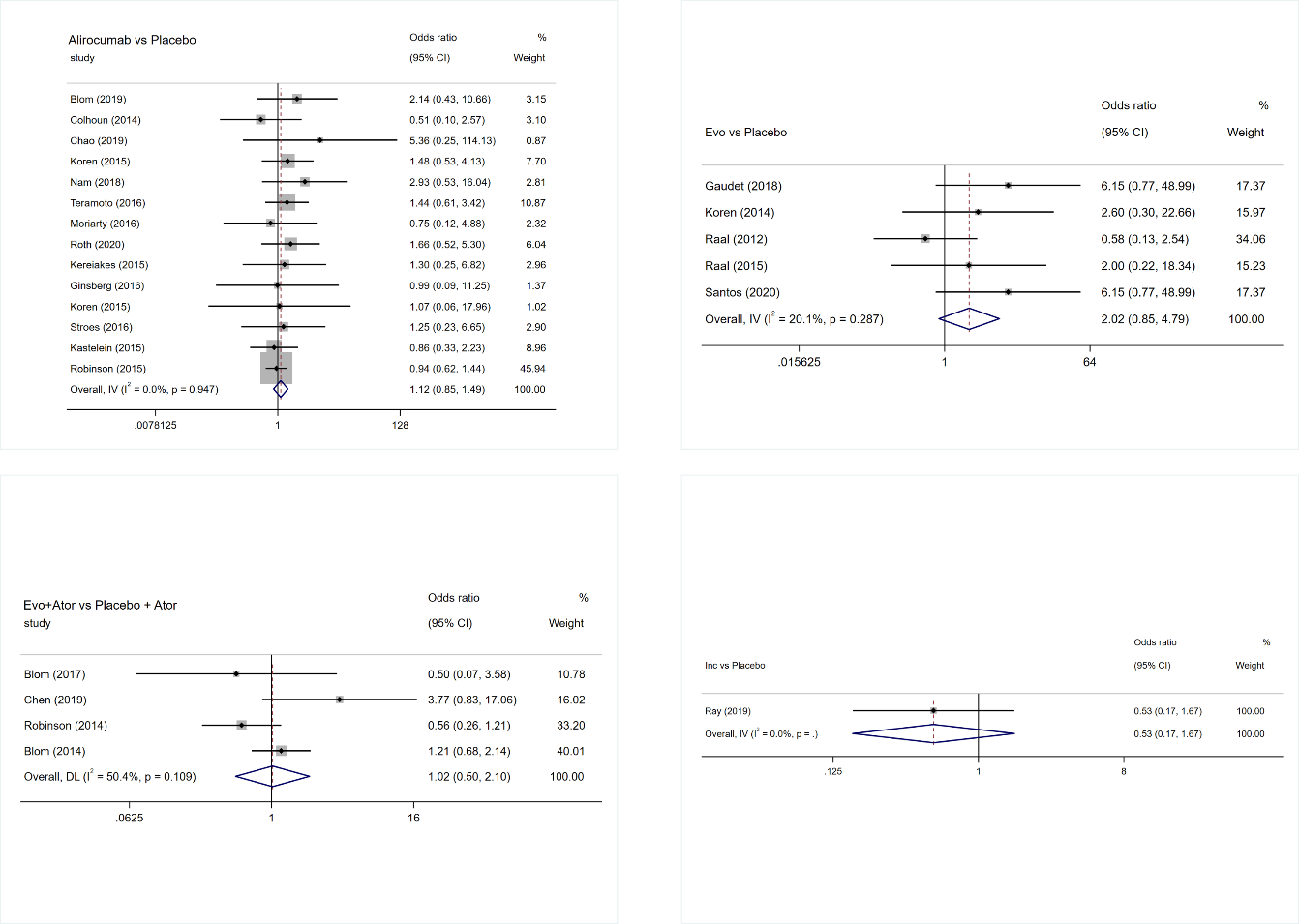

Supplement: Supplementary file 1 [file Datasheet1.docx]
